# CD19 CAR T cells for B cell malignancies: a systematic review and meta-analysis focused on clinical impacts of CAR structural domains, manufacturing conditions, cellular product, doses, patient’s age, and tumor types

**DOI:** 10.1186/s12885-024-12651-6

**Published:** 2024-08-22

**Authors:** Erik Montagna, Najla Santos Pacheco de Campos, Victoria Alves Porto, Giselle Correia Próspero da Silva, Eloah Rabello Suarez

**Affiliations:** 1 Centro Universitário FMABC, Santo André, 09060-870 SP Brazil; 2 Center for Natural and Human Sciences, Federal University of ABC, Santo Andre, 09210-580 SP Brazil; 3 Graduate Program in Medicine - Hematology and Oncology, Federal University of São Paulo, São Paulo, 04023-062 SP Brazil

**Keywords:** Hinge, Transmembrane, Costimulatory domain, CD28, 4-1BB, CD8, CAR T cells manufacturing conditions, CAR T cell dose, Clinical response CAR T cells

## Abstract

CD19-targeted chimeric antigen receptors (CAR) T cells are one of the most remarkable cellular therapies for managing B cell malignancies. However, long-term disease-free survival is still a challenge to overcome. Here, we evaluated the influence of different hinge, transmembrane (TM), and costimulatory CAR domains, as well as manufacturing conditions, cellular product type, doses, patient’s age, and tumor types on the clinical outcomes of patients with B cell cancers treated with CD19 CAR T cells. The primary outcome was defined as the best complete response (BCR), and the secondary outcomes were the best objective response (BOR) and 12-month overall survival (OS). The covariates considered were the type of hinge, TM, and costimulatory domains in the CAR, CAR T cell manufacturing conditions, cell population transduced with the CAR, the number of CAR T cell infusions, amount of CAR T cells injected/Kg, CD19 CAR type (name), tumor type, and age. Fifty-six studies (3493 patients) were included in the systematic review and 46 (3421 patients) in the meta-analysis. The overall BCR rate was 56%, with 60% OS and 75% BOR. Younger patients displayed remarkably higher BCR prevalence without differences in OS. The presence of CD28 in the CAR’s hinge, TM, and costimulatory domains improved all outcomes evaluated. Doses from one to 4.9 million cells/kg resulted in better clinical outcomes. Our data also suggest that regardless of whether patients have had high objective responses, they might have survival benefits from CD19 CAR T therapy. This meta-analysis is a critical hypothesis-generating instrument, capturing effects in the CD19 CAR T cells literature lacking randomized clinical trials and large observational studies.

**Supplementary Information:**

The online version contains supplementary material available at 10.1186/s12885-024-12651-6.

## Introduction

Chimeric antigen receptors (CARs) are artificial cell membrane receptors responsible for immune cell activation. They are constituted by an extracellular binding domain selected against an antigen, usually in the form of a single-chain variable fragment (scFv), a hinge sequence, and a transmembrane domain fused to intracellular costimulatory and stimulatory signaling domains. First-generation CARs had only one CD3ζ chain in the intracellular domain for T cell activation. Second- and third-generation CARs harbor one and two additional intracellular costimulatory domains, respectively, eliciting complete T cell activation. Fourth-generation CARs are based on second or third-generation CARs designed in a vector able to induce the expression of additional transgenic products, constitutively or by induction, such as cytokines or monoclonal antibodies. The CAR expression has been vastly explored in T cells (CAR T cells), and is evolving in other immune cell types, such as NK cells, dendritic cells, and macrophages, ushering in a new era for the treatment of cancer and other diseases [1, 2]. In clinical trials, the main domains constituting the hinge part of a CAR are CD28, CD8 alpha, IgG4, or IgG1, while for the transmembrane domain (TM), CD28 or CD8 alpha are the most applied. The costimulatory domains more extensively applied in the clinical setting are CD28 and 4-1BB. CD28 incorporation into the costimulatory domain of CD19 CAR elicits tumor eradication, glycolysis, effector memory maturation, and T cell exhaustion, whereas 4-1BB signaling induces in vivo T cell persistence, mitochondrial biogenesis, and reprogramming towards a central memory T cell phenotype [3]. Regardless of a few small studies that explored the clinical impact of using different costimulatory domains in the CAR, there is a lack of information about the influence of different hinge or TM domains on the clinical outcomes of patients treated with CAR T cells.

One of the current most effective CAR T cell therapies targets CD19, an antigen expressed by B cells in all stages of development until differentiation in plasmocytes, including B cell malignancies, such as Hodgkin (HL) and non-Hodgkin lymphoma (NHL), acute (ALL) or chronic lymphocytic leukemia (CLL) [4]. All tumor types treated with this therapy had a high initial complete response (CR) rate, but long-term disease-free survival can still be improved [4]The therapeutic success of CAR T cells is sometimes discrepant as it is shaped by several factors, boosting the conduction of a comparative analysis to address the global impact of in vivo and ex vivo conditions that influence CD19 CAR T cell performance in clinical trials.

Here, we analyzed the rates of the primary outcome – defined as best complete response (BCR) – and secondary outcomes defined as 12-month overall survival (OS) and best objective response (BOR) of CD19-positive leukemia or lymphoma patients treated with CD19 CAR T cells containing different hinge, transmembrane (TMD), and costimulatory domains. We have also analyzed the impact of different parameters related to CAR T cell manufacturing conditions, such as the type of interleukin used for CAR T cell expansion, CAR T cells activation method, and cell population transduced with the CAR. We have also evaluated the number of CAR T cell infusions, amount of CAR T cells injected/Kg, CD19 CAR type (name), tumor type, and age. This meta-analysis will be helpful as a hypothesis-generating instrument as it tries to capture effects in the literature that is still recent, lacking randomized clinical trials and large observational studies.

## Methods

### Search strategy

We accomplished a systematic review and meta-analysis according to the PRISMA statement [5, 6], registered on PROSPERO (CRD42022360268). The main study question is the rate of BCR in patients undergoing treatment for B cell malignancies according to the CD19 CAR T cells hinge, transmembrane, and costimulatory domains. The MEDLINE/PubMed database was searched from the inception until August 2021, using the following keywords: “receptors, chimeric antigen“[MeSH Terms] OR (“receptors“[All Fields] AND “chimeric“[All Fields] AND “antigen“[All Fields]) OR “chimeric antigen receptors“[All Fields] OR (“chimeric“[All Fields] AND “antigen“[All Fields] AND “receptor“[All Fields]) OR “chimeric antigen receptor“[All Fields]) AND “CD19“[All Fields].

### Study eligibility criteria

The inclusion criteria were patients with CD19-positive leukemia or lymphoma treated with second or third-generation CD19 CAR T cells. Only studies with original data and in English were included. Grey literature and reference lists from included studies were also considered.

The exclusion criteria were studies with (a) no primary outcome reported, (b) dual CAR, (c) other CAR cells types, such as CAR macrophages, (d) combinations with CAR T cells targeting other molecules or with other targeted or non-targeted therapies, such as hematopoietic stem cell transplant, (e) patients with multiple myeloma and other non-hematological tumors, (f) case series, (g) studies such as meta-analyses, reviews, case reports, protocols, books, letters to the editor, comments or specialists’ opinions, abstracts, and (h) pre-clinical studies. Studies ≤ 10 patients were included in the evidence summary but were excluded from the meta-analysis due to statistical constraints.

### Data extraction

Data extracted comprised the rate of successful outcomes versus the sample included in the study, and BCR was defined as the primary outcome. The secondary outcomes were OS and BOR. For the meta-analysis, categorical covariates were the types of hinge, TM, and costimulatory (costimulation) domains in the CAR, CAR T cell manufacturing conditions, such as the interleukin used for CAR T cell expansion, CAR T cells activation method, and cell population transduced with the CAR – PBMCs or other specific subsets – (CAR T cell type), as well as the CD19 CAR type (CAR name), and tumor type. Numerical covariates were patient age, number of CAR T cells injected/Kg, and the number of CAR T cells infusions.

Two independent investigators (ERS and NSPC) screened titles and abstracts with ties resolved by a third person (VAP). Three authors (NSPC, VAP, GCPS) independently performed the full-text review and extracted the data, and ERS resolved disagreements.

### Data syntheses

The data was presented in a summary of evidence and synthesized as forest plots, with studies ordered by publication year. All methodological details of the meta-analysis were included in the Supplementary Methods.

### Risk of bias assessment

Risk of bias assessment adopted the Modified Institute of Health Economics Tool for bias analysis [7] and was performed independently by three authors (NSPC, VAP, GCPS).

### Statistical analysis

Statistical analysis was performed with RStudio version 1.1.383 (The R Foundation for Statistical Computing, Vienna, Austria), using *meta* and *metafor* packages [8, 9].

## Results

Fifty-six studies were included in the systematic review with a total of 3493 patients, 2904 treated with CAR T, and 2809 patients analyzed for rate estimation of BCR. Of these patients, 1440 presented a CR, and 1587 had an objective response (OR). We have also evaluated 12 months-OS, having 42 studies with a total of 2992 patients included, 2479 patients treated with CAR T, and 2393 patients analyzed, of whom 1567 were alive at 12 months.

**Fig. 1:**
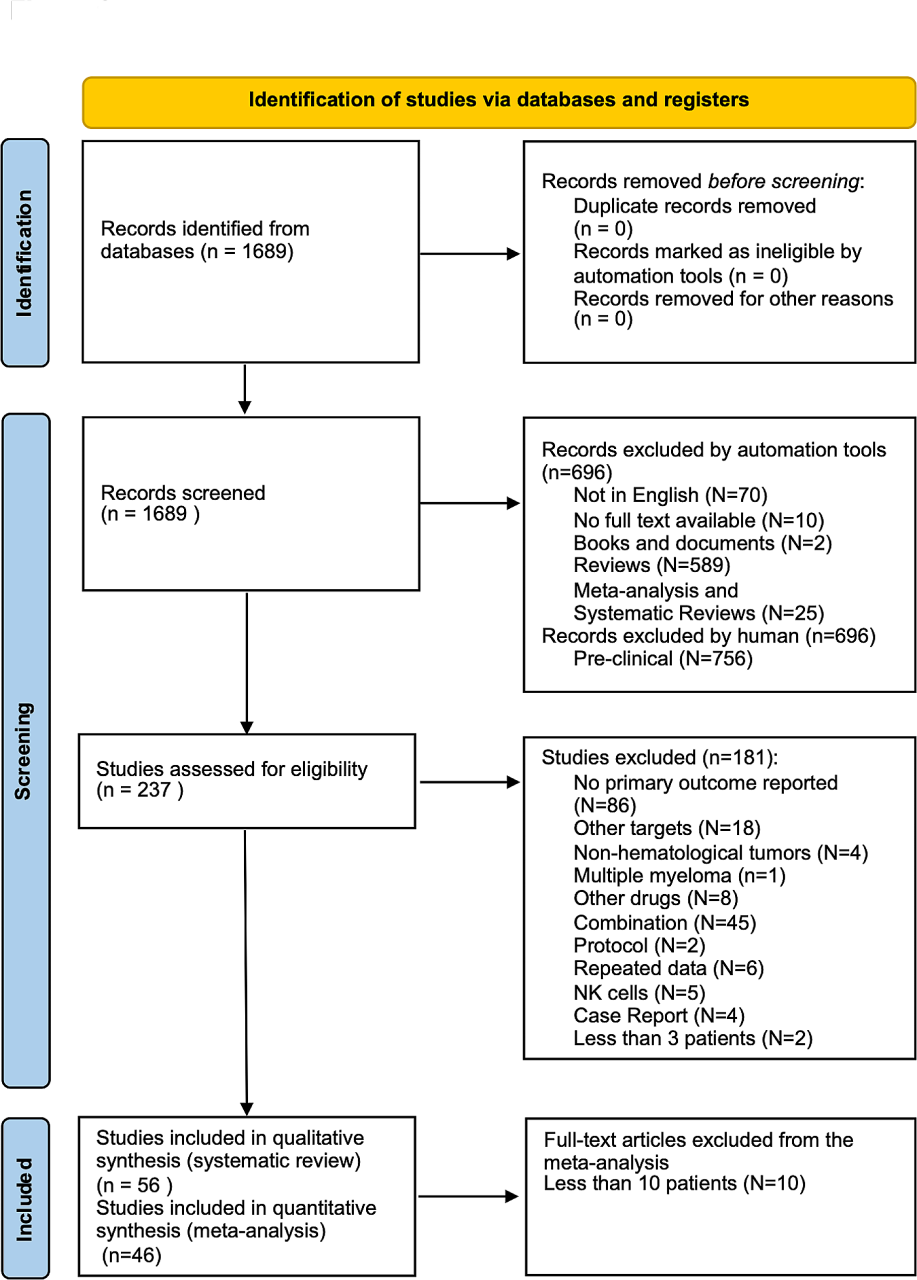
Flow chart for eligibility assessment according to PRISMA guidelines. Based on model reference(Page et al., 2021a). For more information, visit: http://www.prisma-statement.org/

A total of 46 studies with more than or equal to 10 patients were included in the meta-analysis involving 3421 patients, of whom 2837 were treated with CAR T and 2746 patients analyzed for rate estimation of the primary outcome BCR, being 1251 patients presenting CR and 1571 presenting OR, one of the secondary outcomes evaluated. For the other secondary outcome assessed, OS, we had 37 studies with 2949 patients, 2439 patients treated with CAR T, and 2356 patients analyzed for OS, of whom 1547 were alive at 12 months. The PRISM flow diagram is present in Fig. 1, and the summary of evidence in Table 1.

**Table 1:** Evidence summary

Study Ref Number	Study/ Year	Cell population transduced or transfected with the CAR	CAR hinge domain	CAR transmembrane domain	CAR costimulatory domain	Anti-CD19 CAR Type (Name)	Interleukin used for CAR T cell expansion in vitro	CAR T cells activation method	Number of CART cells injected/ kg	Number of CAR T cell infusions	Age (median/ range)	Number of Treated Patients	Number of Patients Analyzed for Response	Tumor Type	Best Objective Response (%)	Best Complete Response (%)	Overall Survival 12 months (%)
2011BRE	Brentjens et al. (2011) [10] #	PBMCs	IgG4	CD28	CD28	MDACC	IL-2	Beads Anti-CD3/CD28	≥ 10e8	Variable	68 (51–73)	9	8	ALL + CLL	57	0	78
2011KA	Kalos et al. (2011) [11] #	PBMCs	CD8	CD8	4-1BB	Tisa-cel	NF	Beads Anti-CD3/CD28	5 × 10e6-9,9 × 10e7	1	69 (64–77)	3	3	CLL	100	75	NF
2012KO	Kochenderfer et al. (2012) [12]	PBMCs	CD28	CD28	CD28	Axi-cel	IL-2	Anti-CD3 mAb	5 × 10e6-9,9 × 10e7	1	56 (48–63)	8	7	NHL + CLL	86	14	NF
2014BR	Brentjens et al. (2014) [13] #	PBMCs	IgG4	CD28	CD28	MDACC	IL-2	Beads Anti-CD3/CD28	5 × 10e6-9,9 × 10e7	2	52 (23–66)	5	5	ALL	100	100	NF
2015 K	Kochenderfer et al. (2015) [14]	PBMCs	CD8	CD8	4-1BB	Tisa-cel	IL-2	Anti-CD3 mAb	5 × 10e6-9,9 × 10e7	1	51 (30–68)	15	13	NHL + CLL	92	62	53
2015LE	Lee et al. (2015) [15]	PBMCs	CD8	CD8	4-1BB	Tisa-cel	IL-2	Anti-CD3 mAb	1–4,9 × 10e6	Variable	15 (5–27)	21	21	ALL	67	67	NF
2016BH	Bhoj et al. (2016) [16]	PBMCs	CD8	CD8	4-1BB	Tisa-cel	NF	Beads Anti-CD3/CD28	1–4,9 × 10e6	NF	25 (5–64)	16	16	CLL + ALL + NHL	100	100	NF
2016Keb	Kebriaei et al.(2016) [17] †	PBMCs	IgG4	CD28	CD28	MDACC	IL-2 + others*	Anti-CD3 mAb	≥ 10e8	1	41 (21–61)	26	26	NHL + ALL	100	35	69
2016RAM	Ramos et al. (2016) [18] †	PBMCs	IgG1	CD28	1st gen	Others	IL-2 + others*	Anti-CD3 mAb	≥ 10e8	1	59 (43–75)	16	16	NHL + CLL	19	13	NF
2016TUR	Turtle et al. (2016) [19] †	Specific subsets	IgG4	CD28	4-1BB	JCAR014	IL-2	Beads Anti-CD3/CD28	1–4,9 × 10e6	1	57 (22–70)	64	60	NHL	63	33	19
2017GAR	Gardner et al. (2017) [20]	Specific subsets	IgG1	CD28	4-1BB	Others	IL-2 + others*	Beads Anti-CD3/CD28	1–4,9 × 10e6	2	12 (1.3–25.3)	43	43	ALL	95	93	70
2017HU	Hu et al. (2017) [21]	PBMCs	CD8	CD8	4-1BB	Tisa-cel	IL-2	Beads Anti-CD3/CD28	1–4,9 × 10e6	≥ 3 infusions	32 (7–57)	15	15	ALL	73	40	NF
2017LOCK	Locke et al. (2017) [22] #	PBMCs	CD28	CD28	CD28	Axi-cel	IL-2	Anti-CD3 mAb	1–4,9 × 10e6	1	59 (29–69)	7	7	NHL	71	57	86
2017NEE	Neelapu et al. (2017) [23]	PBMCs	CD28	CD28	CD28	Axi-cel	IL-2	Beads Anti-CD3/CD28	1–4,9 × 10e6	1	58 (23–76)	101	101	NHL	81	53	61
2017SCH	Schuster et al. (2017) [24]	PBMCs	CD8	CD8	4-1BB	Tisa-cel	NF	Beads Anti-CD3/CD28	5 × 10e6-9,9 × 10e7	1	57 (25–77)	28	28	NHL	64	57	75
2017TUR	Turtle et al. (2017) [25]	Specific subsets	IgG4	CD28	4-1BB	JCAR014	IL-2	Beads Anti-CD3/CD28	1–4,9 × 10e6	1	61 (40–73)	24	19	CLL	74	21	58
2018ENB	Enblad et al. (2018) [26]	PBMCs	CD28	CD28	3rd gen	Others	IL-2	Anti-CD3 mAb	≥ 10e8	1	61 (24–71)	15	15	NHL + ALL	40	40	33
2018GEYE	Geyer et al. (2018) [27] #	PBMCs	CD28	CD28	CD28	Axi-cel	IL-2	Beads Anti-CD3/CD28	5 × 10e6-9,9 × 10e7	2	58 (45–70)	8	8	CLL	38	25	100
2018JAC	Jacoby et al. (2018) [28]	PBMCs	CD28	CD28	CD28	Axi-cel	IL-2	Anti-CD3 mAb	1–4,9 × 10e6	1	11 (5–48)	20	20	ALL	90	90	90
2018MAU	Maude et al. (2018) [29]	PBMCs	CD8	CD8	4-1BB	Tisa-cel	NF	Beads Anti-CD3/CD28	1–4,9 × 10e6	1	11 (3–23)	75	75	ALL	80	60	76
2018PARK	Park et al. (2018) [30]	PBMCs	CD28	CD28	CD28	Axi-cel	NF	Beads Anti-CD3/CD28	1–4,9 × 10e6	≥ 3 infusions	44 (23–74)	53	52	ALL	87	62	55
2018ROSS	Rossi et al. (2018) [31]	PBMCs	CD28	CD28	CD28	Axi-cel	IL-2	Beads Anti-CD3/CD28	NF	NF	53 (28,6–67,8)	20	20	NHL	70	50	NF
2018SVO	Svoboda et al. (2018) [32] #	PBMCs	CD8	CD8	4-1BB	Tisa-cel	NF	Beads Anti-CD3/CD28	≤ 9.9 × 10e5	≥ 3 infusions	23 (21–42)	4	4	HL	50	25	NF
2018WEN	Weng et al. (2018) [33] #	PBMCs	CD28	CD28	3rd gen	Others	NF	Beads Anti-CD3/CD28	≤ 9.9 × 10e5	≥ 3 infusions	20 (15–34)	3	3	ALL	100	100	NF
2019CUR	Curran et al. (2019) [34]	PBMCs	IgG4	CD28	CD28	MDACC	NF	Beads Anti-CD3/CD28	1–4,9 × 10e6	≥ 3 infusions	13 (1-22.5)	25	24	ALL	75	67	44
2019GHO	Ghorashian et al. (2019) [35]	PBMCs	CD8	CD8	4-1BB	Others	NF	Beads Anti-CD3/CD28	1–4,9 × 10e6	1	9 (1.35–19.28)	14	14	ALL	86	71	57
2019HA	Hay et al. (2019) [36]	Specific subsets	IgG4	CD28	4-1BB	JCAR014	IL-2	Beads Anti-CD3/CD28	1–4,9 × 10e6	Variable	39 (20–76)	53	53	ALL	85	85	NF
2019HI	Hirayama et al. (2019) [37]	Specific subsets	IgG4	CD28	4-1BB	JCAR014	IL-2	Beads Anti-CD3/CD28	1–4,9 × 10e6	1	58 (52–63)	48	47	NHL	49	45	NF
2019HI	Hirayama et al. (2019) [38]	Specific subsets	IgG4	CD28	4-1BB	JCAR014	IL-2	Beads Anti-CD3/CD28	1–4,9 × 10e6	1	56 (51–62)	21	21	NHL	57	57	NF
2019Lock	Locke et al. (2019) [39]	PBMCs	CD28	CD28	CD28	Axi-cel	IL-2	Anti-CD3 mAb	1–4,9 × 10e6	1	52 (34–64)	108	101	NHL	83	58	NF
2019 Ma	Ma et al. (2019) [40] #	Specific subsets	CD28	CD28	4-1BB	Others	IL-2	Beads Anti-CD3/CD28	5 × 10e6-9,9 × 10e7	1	6 (3–13)	10	8	ALL	75	25	20
2019Schuster	Schuster et al. (2019) [41]	PBMCs	CD8	CD8	4-1BB	Tisa-cel	NF	Anti-CD3 mAb	≥ 10e8	1	56 (22–76)	111	93	NHL	51	40	25
2019YING	Ying et al. (2019) [42]	PBMCs	CD8	CD8	4-1BB	Tisa-cel	IL-2	Beads Anti-CD3/CD28	≥ 10e8	Variable	48 (76 − 24)	25	25	HL + NHL	68	28	NF
2019ZHANG	Zhang et al. (2019) [43] #	PBMCs	CD8	CD8	4-1BB	Tisa-cel	IL-2	Beads Anti-CD3/CD28	NF	2	48 (29–59)	4	4	ALL	75	75	NF
2020ABR	Abramson et al. (2020) [44] †	Specific subsets	IgG4	Others/ Mixed	4-1BB	Others	NF	Beads Anti-CD3/CD28	Variable	Variable	63 (54–70)	294	256	NHL	73	53	58
2020AN	An et al. (2020) [45]	PBMCs	IgG4	CD28	3rd gen	Others	IL-2	Beads Anti-CD3/CD28	1–4,9 × 10e6	1	22 (3–72)	47	47	ALL	NF	81	100
2020BEN	Benjamin et al. (2020) [46]†	PBMCs	CD8	CD8	4-1BB	Others	IL-2	Beads Anti-CD3/CD28	Variable	Variable	22 (14–39)	21	21	ALL	7	19	38
2020CAP	Cappell et al. (2020) [47] †	PBMCs	CD28	CD28	CD28	Axi-cel	IL-2	Anti-CD3 mAb	1–4,9 × 10e6	1	54 (26–68)	43	43	NHL + CLL	81	58	77
2020chen0	Chen et al. (2020) [48] †	PBMCs	Two arms below	Two arms below	Two arms below	Two arms below	IL-2	Beads Anti-CD3/CD28	≤ 9.9 × 10e5	1	21 (2–55)	35	35	ALL	86	88	46
2020chen1	First arm#	PBMCs	CD28	CD28	CD28	Axi-cel	IL-2	Beads Anti-CD3/CD28	≤ 9.9 × 10e5	1	NF	6	6	ALL	NF	67	67
2020chen2	Second arm	PBMCs	CD8	CD8	4-1BB	Tisa-cel	IL-2	Beads Anti-CD3/CD28	≤ 9.9 × 10e5	1	NF	26	26	ALL	NF	92	46
2020FREY	Frey et al. (2020) [49]†	PBMCs	CD8	CD8	4-1BB	Tisa-cel	IL-7 + IL-15	Beads Anti-CD3/CD28	Variable	≥ 3 infusions	61.3 (48.8–76.1)	38	32	CLL	43	28	74
2020GU	Gu et al. (2020) [50]	PBMCs	CD8	CD28	4-1BB	Others	IL-2	Beads Anti-CD3/CD28	5 × 10e6-9,9 × 10e7	1	18 (3–52)	20	20	ALL	90	90	40
2020JA	Jacobson et al. (2020) [51]	PBMCs	CD28	CD28	CD28	Axi-cel	IL-2	Beads Anti-CD3/CD28	1–4,9 × 10e6	1	62 (21–79)	122	116	NHL	NF	70	70
2020LIU	Liu et al. (2020) [52]	PBMCs	CD28	CD28	CD28	Axi-cel	IL-2	Irradiated feeder cells comb	1–4,9 × 10e6	Variable	60 (47–70)	11	11	NHL + CLL	100	64	NF
2020NA	Nastoupil et al. (2020) [53]	PBMCs	CD28	CD28	CD28	Axi-cel	IL-2	Beads Anti-CD3/CD28	1–4,9 × 10e6	1	60 (21–83)	275	275	NHL	NF	64	82
2020PAS	Pasquini et al. (2020) [54]	PBMCs	CD8	CD8	4-1BB	Tisa-cel	NF	Beads Anti-CD3/CD28	Variable	1	39 (0,41–88)	410	410	NHL + ALL	62	39	74
2020SE0	Sesques et al. (2020) [55] †	PBMCs	Two arms below	Two arms below	Two arms below	Two arms below	IL-2	Two arms below	1–4,9 × 10e6	1	59 (27–75)	61	59	NHL	63	48	18
2020SE1	First arm	PBMCs	CD8	CD8	4-1BB	Tisa-cel	IL-2	Beads Anti-CD3/CD28	1–4,9 × 10e6	1	62 (28–75)	33	31	NHL	61	48	39
2020SE2	Second arm	PBMCs	CD28	CD28	CD28	Axi-cel	IL-2	Anti-CD3 mAb	1–4,9 × 10e6	1	59 (27–75)	28	28	NHL	64	46	61
2020WANG	Wang et al. (2020) [56]	PBMCs	CD28	CD28	CD28	Axi-cel	IL-2	Anti-CD3 mAb	1–4,9 × 10e6	1	65 (38–79)	68	60	NHL + ALL	93	67	72
2020ZHO	Zhou et al. (2020) [57]	PBMCs	CD8	CD28	Others/4th gen	Others	NF	Beads Anti-CD3/CD28	≤ 9.9 × 10e5	1	50 (31–77)	21	21	NHL	67	43	76
2021BAI	Baird et al. (2021) [58]	Specific subsets	CD28	CD28	CD28	Axi-cel	IL-2	Beads Anti-CD3/CD28	1–4,9 × 10e6	1	56 (21–76)	41	41	NHL	97	66	68
2021GA	Gauthier et al. (2021) [59]	Specific subsets	IgG4	CD28	4-1BB	JCAR014	IL-7 + IL-15	Beads Anti-CD3/CD28	1–4,9 × 10e6	2	58 (23–73)	44	44	CLL + ALL + NHL	32	22	NF
2021IAC	Iacoboni et al. (2021) [60]	PBMCs	CD8	CD8	4-1BB	Tisa-cel	NF	Beads Anti-CD3/CD28	≥ 10e8	1	60 (52–67)	75	75	NHL	60	32	87
2021MI	Mian et al. (2021) [61]	PBMCs	CD28	CD28	CD28	Axi-cel	IL-2	Beads Anti-CD3/CD28	1–4,9 × 10e6	1	63 (25–77)	27	38	NHL	85	48	37
2021SHA	Shah et al. (2021) [62]	PBMCs	CD28	CD28	CD28	Others	IL-2	Anti-CD3 mAb	1–4,9 × 10e6	1	46 (18–77)	45	45	ALL	69	53	51
2021TA	Tan et al. (2021) [63]	PBMCs	CD8	CD8	4-1BB	Tisa-cel	IL-2	Beads Anti-CD3/CD28	1–4,9 × 10e6	1	8 (1–13)	12	12	ALL	92	92	17
2021WANG	Wang et al. (2021) [64]	PBMCs	CD8	CD8	4-1BB	Others	IL-7 + IL-15	Anti-CD3 mAb	1–4,9 × 10e6	1	12 (0–18)	24	24	ALL	83	83	63
2021YING	Ying et al. (2021) [65]	Specific subsets	IgG4	Others/ Mixed	4-1BB	Others	NF	Beads Anti-CD3/CD28	≥ 10e8	1	56 (18–75)	59	58	NHL	76	52	58

†Studies with two or more arms; *IL-21/IL-15 or IL-7; NF, data not found; HL, Hodgkin lymphoma; NHL, Non-Hodgkin lymphoma; ALL, Acute lymphocytic leukemia; CLL, Chronic lymphocytic leukemia; Axi-cel, Axicabtagene citoleucel (Yescarta^®^, Kite Pharma) or KTE-C19,; Tisa-cel, Tisagenlecleucel (Kymriah^®^, Novartis) or CTL019, MDACC: MD Anderson Cancer Center anti-CD19 CAR product; JCAR014: Juno Therapeutics Anti-CD19 CAR product

### Meta-analysis

#### General clinical responses of CD19 CAR T therapy

**Fig. 2:**
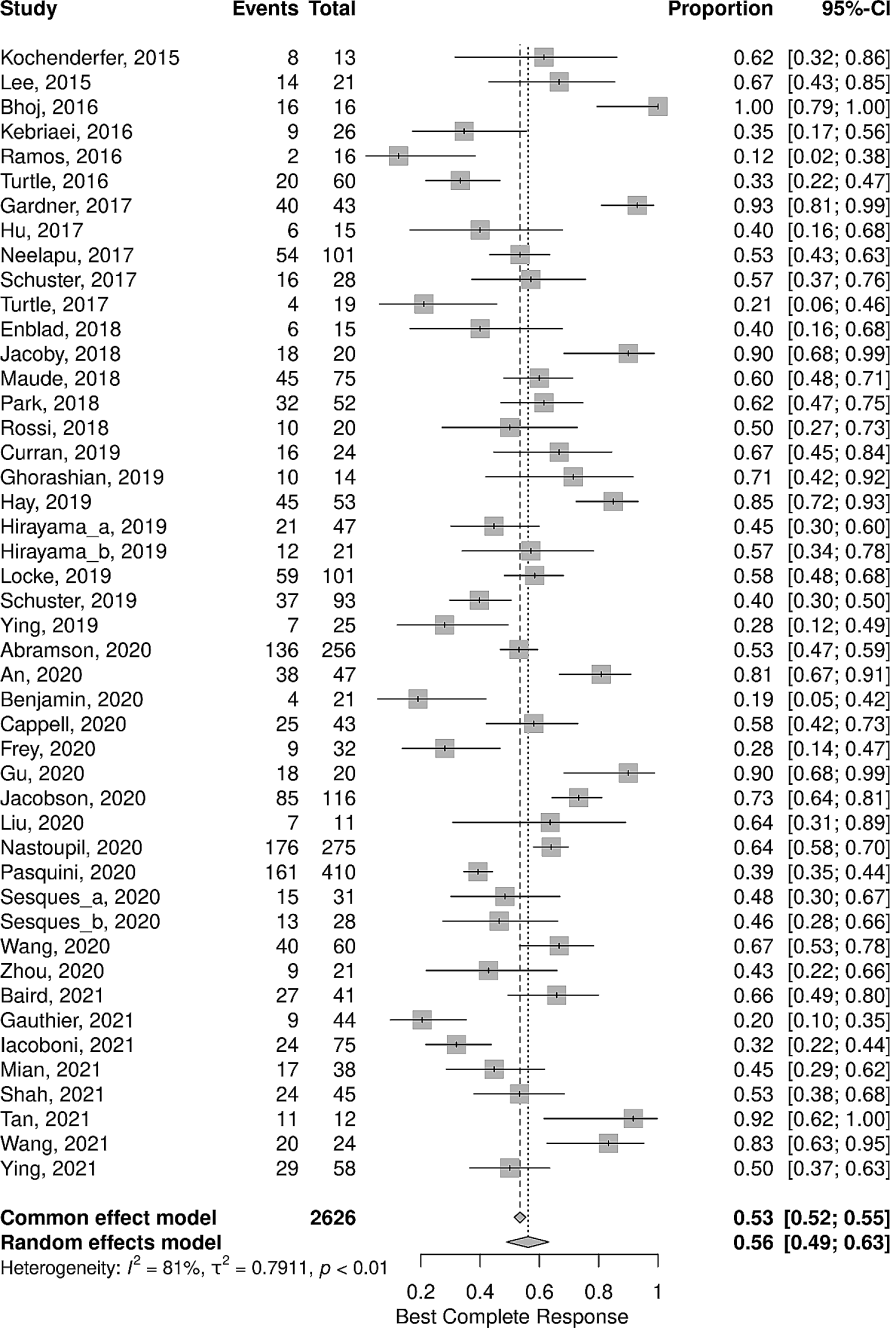
The Forest Plot represents the overall rate of the primary outcome Best Complete Response (BCR) of patients treated with CD19 CAR T therapy based on the studies included in the meta-analysis

**Fig. 3:**
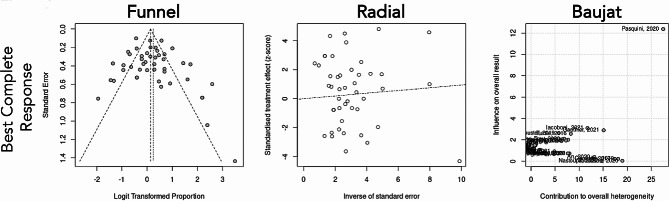
Funnel, Baujat, and Radial plots showing the heterogeneity observed for the primary outcome Best Complete Response (BCR) of patients treated with CD19 CAR T therapy based on the studies included in the meta-analysis

The general proportion of BCR was 56% (95%CI: 49 – 63%), the I^2^ was 81%, and the τ^2^ was 0.7911 indicating a large between-study variance (Fig. 2). However, it equals or exceeds 50% in 28 of 46 studies (Fig. 2). Table 2 summarizes meta-analysis data for primary outcome BCR (also presented in full version with references as Suppl. Table 1). The bias assessment is presented in Fig. 3.

**Table 2:** Best complete response subgroup analysis

Variable	Proportion per subgroup	95% CI	I^2^ (%)
General	0.56	0.49–0.63	81
Age			
< 18	0.79	0.65–0.89	64
> 18	0.51	0.43–0.57	82
Interleukin used for CAR T cell expansion			
IL-2	0.58	0.50–0.66	76
Others/ Not mentioned	0.54	0.43–0.65	72
CAR T Cells Activation Method			
Anti-CD3 mAb	0.55	0.43–0.67	71
Anti-CD3/CD28 beads	0.56	0.47–0.65	84
Number CAR T cells injected/ kg			
1 to 4,9 × 10e6	0.63	0.55–0.71	77
5 × 10e6 to 9,9 × 10e7	0.71	0.25–0.95	62
≥ 10e8	0.36	0.28–0.46	38
Variable	0.40	0.25–0.56	80
Number of CAR T cells infusions			
One infusion	0.55	0.48–0.62	81
Two infusions	0.65	0.00–1.0	97
≥ 3 infusions	0.50	0.25–0.74	74
Variable	0.61	0.33–0.83	79
Cell population transduced or transfected with the CAR			
PBMCs	0.57	0.49–0.64	80
CD4/CD8 1:1 or CD8 + only or specific subsets	0.54	0.34–0.73	87
Anti-CD19 CAR Type (Name)			
Axicabtagene citoleucel (Yescarta) (Axi-cel) KTE-C19	0.62	0.56–0.67	52
Tisagenlecleucel (CTL019) (Kymriah) (Tisa-cel)	0.53	0.38–0.67	66
JCAR014	0.44	0.20–0.71	88
Others	0.60	0.40–0.78	82
CAR Hinge Domain			
CD8	0.56	0.42–0.70	75
CD28	0.60	0.55–0.66	52
IgG4	0.50	0.35–0.66	85
CAR Transmembrane Domain			
CD8	0.54	0.40–0.68	73
CD28	0.58	0.48–0.67	80
CAR Costimulatory Domain			
CD28	0.60	0.54–0.66	55
4-1BB	0.56	0.44–0.67	82
Tumor Type			
NHL	0.51	0.45–0.57	75
ALL	0.73	0.60–0.83	77

The general proportion of OS was 60% (95%CI: 53 – 67%), the I^2^ was 87%, and the τ^2^ was 0.5642 (Suppl. Figure 1 and Suppl. Table 2) indicating a moderate between study variance. The overall rate of BOR with CD19 CAR T therapy was 75% (95% CI: 68 – 82%, I^2^ = 78%) with a very high between-study variance (τ2 = 1.2262) and rates equal to or above 50% in 40 of 46 studies (Suppl. Figure 2 and Suppl. Table 3). Together, these data indicate substantial heterogeneity. The bias assessment for OS and BOR are also presented in Suppl. Figure 3. All the other forest plots are presented as Suppl. Figure 4 to 39.

### Sensitivity -analysis

#### Age

Patients under 18 years old had a 79% BCR (95%CI: 65-89%, I^2^:64%), 62% OS (95%CI: 41-80%, I^2^:73%) and 84% BOR (95%CI: 75-90%, I^2^:31%) (Suppl Figs. 4, 5 and 6, respectively). Patients above 18 years old presented a 51% BCR (95%CI: 43 − 57%, I^2^:82%), 60% OS (95%CI: 52- 67%, I^2^:88%) and 73% BOR (95%CI: 64-81%, I^2^:79%) (Suppl Figs. 4, 5 and 6, respectively).

#### CD19 CAR T cells manufacturing conditions

Considering interleukin used for CAR T cell expansion, when IL-2 was used we found 58% BCR (95%CI: 50-66%, I^2^:76%), 56% OS (95%CI: 45-66%, I^2^:86%) and 79% BOR (95%CI: 68-87%, I^2^:70%) (Suppl Figs. 7, 8 and 9, respectively). When other interleukins were applied, we had a 54% BCR (95%CI: 43-65%, I^2^:72%), 63% OS (95%CI: 50-75%, I^2^:91%), and 73% BOR (95%CI: 64-80%, I^2^:71) (Suppl Figs. 7, 8 and 9, respectively).

The BCR (Suppl Fig. 10), OS (Suppl Fig. 11), and BOR (Suppl Fig. 12) proportions were similar for activation and expansion of CAR T cells with anti-CD3/CD28 beads or anti-CD3 mAb. Considering the cell population transduced with the CAR, we have found similar BCR (Suppl Fig. 13) and BOR (Suppl Fig. 14) rates when using full PBMCs or CD4/CD8 1:1, CD8 only, or other specific subsets. OS rate was higher when using full PBMCs (61%; 95%CI: 53–73%, I^2^: 86%) compared to 55% for CD4/CD8 1:1, CD8 only, or other specific subsets (55%; 95%CI: 35–73%, I^2^: 86%) (Suppl Fig. 15).

#### Number of T cells injected into the patients/Kg

Patients treated with doses between 1 and 4.9 million cells/ kg per injection had BCR rates of 63% (95%CI: 55-71%, I^2^:77%), 60% OS (95%CI: 50-69%, I^2^:85%), and 83% BOR (95%CI: 76-88%, I^2^:74%) (Suppl Figs. 16, 17 and 18, respectively). The 5 to 99 million cells/kg group had only three studies and was not considered for comparison (71% BCR; 95%CI: 25-95%, I^2^:62%; 58% OS; 95%CI: 21-88%, I^2^:66%, and 83% BOR, 95%CI:29–98%, I^2^: 64) (Suppl Figs. 16, 17 and 18, respectively). Doses superior to 100 million cells/kg showed lower BCR (36%; 95%CI: 28–46%, I^2^:38%), OS (56%, 95%CI: 25–83%, I^2^:94%) and BOR rates (64%, 95%CI: 32-87%, I^2^:69%) (Suppl Figs. 16, 17 and 18, respectively).

#### Number of CAR T cell infusions in the patients

The proportions for a single cell injection were 55% for BCR (95%CI:48-62%, I^2^: 81%), 61% for OS (95%CI:52-69%, I^2^: 88%) and 78% for BOR (95%CI:69-85%, I^2^: 77%) (Suppl Figs. 19, 20 and 21, respectively). For two infusions, the number of studies was meager (65% BCR; 95%CI: 0-100, I^2^: 97%; 70% OS, 95%CI:55-82%, I^2^: not applicable) (Suppl Figs. 19, 20 and 21, respectively). Studies with three or more infusions showed a 50% BCR rate (50%; 95%CI: 25–74%, I^2^: 74%) and 72% BOR (95%CI: 40–91%, I^2^: 81%) (Suppl Fig. 19, and 21, respectively). For OS, the number of studies was also meager (58% OS, 95%CI: 29–83%, I^2^: 66%) (Suppl Fig. 20).

#### CD19 CAR T cell products

For Axicabtagene ciloleucel (Axi-cel), we have found a 62% BCR (95%CI: 56–67%, I^2^: 52%), 68% OS (95%CI: 59–77%%, I^2^: 80%) and 86% BOR rates (95%CI: 78–91%, I^2^: 46%) (Suppl Figs. 22, 23 and 24, respectively). Tisagenlecleucel (Tisa-cel) showed 53% BCR (95%CI:38–67%, I^2^: 66%), 61% OS (95%CI:42–76%, I^2^: 92%) and 70% BOR rates (95%CI:59–79%, I^2^: 55%) (Suppl Figs. 22, 23 and 24, respectively). Other CD19 CAR T products more recently tested had a 60% BCR (95%CI:40–78%, I^2^:82%), 57% OS (95%CI:52–62%, I^2^:40%), and 67% BOR rates (95%CI:44–86%, I^2^:80%) (Suppl Figs. 22, 23 and 24, respectively).

#### CAR hinge domain

When CD28 was used to construct the CAR hinge domain, we had a 60% BCR (95%CI:55–66%, I^2^: 52%), 65% OS (95%CI:55–74%, I^2^: 81%) and 83% BOR rates (95%CI:73–90%, I^2^: 66%) (Suppl Figs. 25, 26 and 27, respectively). For CD8, we observed 56% BCR (95%CI:42–70%, I^2^: 75%), 59% OS (95%CI:46–71%, I^2^: 89%), and 71% BOR (95%CI:58–82%, I^2^: 66%) (Suppl Figs. 25, 26 and 27, respectively). IgG4 resulted in 50% BCR (95%CI:35–66%, I^2^: 85%), 50% OS (95%CI:32–59%, I^2^: 84%) and 71% BOR (95%CI: 54–83%, I^2^: 79%) (Suppl Figs. 25, 26 and 27, respectively).

#### CAR transmembrane domain

When the CD28 transmembrane domain was used to build the CAR, we found a 58% BCR (95%CI:48–67%, I^2^: 80%), 61% OS (95%CI:51–70%, I^2^: 85%) and 79% BOR (95%CI:69–86%, I^2^: 80%) (Suppl Figs. 28, 29 and 30, respectively). CD8 alpha in the transmembrane resulted in 54% BCR (95%CI:40–68%, I^2^: 73%), 59% OS (95%CI:45–72%, I^2^: 90%) and 70% BOR (95%CI:55–82%, I^2^: 67%) (Suppl Figs. 28, 29 and 30, respectively).

#### CAR costimulatory domain

The CD28 costimulatory domain in the CAR resulted in 60% BCR (95%CI:54–66%, I^2^: 55%), 66% OS (95%CI:57–74%, I^2^: 79%) and 85% BOR rates (95%CI:78–91%, I^2^: 45%), while for 4-1BB we had 56% BCR (95%CI:44–67%, I^2^: 82%), 56% OS (95%CI:45–66%, I^2^: 89%) and 71% BOR (95%CI:61–79%, I^2^: 76%) (Suppl Figs. 31, 32 and 33, respectively).

#### Tumor type

Patients with ALL achieved 73% BCR (95%CI:60–83%, I^2^: 77%), 57% OS (95%CI:45–68%%, I^2^: 67%), and 80% BOR (95%CI:66–89%, I^2^: 64%) %) (Suppl Figs. 34, 35 and 36, respectively), while for NHL, the general BCR was 51% (95%CI:45–57%, I^2^: 75%), 59% OS (95%CI:46–72%, I^2^: 92%) and 71% BOR (95%CI:63–78%, I^2^: 74%) (Suppl Figs. 34, 35 and 36, respectively).

### Meta-regression

The meta-regression showed that the group aged above 18 presented a low but significant amount of heterogeneity explained by this variable (H^2^ = 7.5535) and that the moderator is inversely related to BCR, suggesting that the effect size favors the younger patient (estimate= -1.3211; *p* = 0.005). Also, costimulation based on CD28 and third-generation CD28/4-1BB presents a small amount of heterogeneity explained (H^2^ = 9.1079), but both were statistically significant moderators (*p* = 0.0391 and *p* = 0.0493, respectively). For BOR, the attributable heterogeneity for costimulatory domains was H^2^ = 7.5535, and CD28 and 4-1BB were significant for this observation (*p* = 0.0047 and *p* = 0.0355). The attributable heterogeneity for the CAR T cell product was small (H^2^ = 7.4956); however, there was an inverse effect for Tisa-cel and JCAR014 as moderators (*p* = 0.0336 and *p* = 0.0097). Finally, for OS, the attributable heterogeneity for the CAR T cell product was H^2^ = 6.0343, and only the treatment with JCAR014 presented an inverse and statistically significant moderator effect (*p* = 0.0215).

### Risk of bias assessment

A predominant low risk of bias was assessed for the primary and the secondary outcomes, presented in Suppl. Figures 37, 38, and 39, respectively.

## Discussion

The pooled 56%BCR found for all CD19 CAR T therapies evaluated herein, with a 60% OS and 75% BOR, corroborates the results found in most CD19 CAR T clinical trials [66]. However, among the studies included in this meta-analysis, there are also some outliers, such as one published by Ramos et al. (2016), showing only 13% BCR and 19% BOR (*N* = 16 patients, no OS reported), that can be explained by the employment of a first-generation CAR, which usually fails to reach effective antitumor responses [67, 68].For comparison, a meta-analysis focused on DLBCL conducted in 2022 by Ying and collaborators showed a similar pooled 63% OS rate and 74% BOR, diverging only by an expressively lower 48% BCR [69].Additionally, another meta-analysis published in 2021 by Aamir et al., focused on ALL patients, reported an 82% BCR rate. Neither OS nor BOR were reported in this study for comparison [70]. The difference in pooled BCR from these two studies compared with ours can be explained, at least in part, by the mixed tumor types included in our study, such as ALL, CLL, and other NHL subtypes. When we compared ALL and NHL in our sensitivity analysis for tumor type, the most expressive differences between them were also found for BCR (73 versus 51%), followed by BOR (80 versus 71%) rates, while both tumors resulted in similar OS rates (59 versus 57%). Our data also suggest that regardless of whether patients have had high objective responses or not, they might have survival benefits from CD19 CAR T therapy.

Among the CAR T manufacturing conditions evaluated herein, the cell populations chosen to build the CAR product and the cytokine used for T cell expansion promoted the most relevant differences for the clinical outcomes analyzed, mainly for OS. PBMCs had higher OS but similar BOR and BCR rates compared to CD4/CD8 1:1 clustered with CD8 and other specific subsets for analysis. The clustering of CD4/CD8 1:1, CD8 alone, or others might have influenced the results obtained since there is pre-clinical and clinical evidence that CD4:CD8 1:1 seems to outperform other populations. However, we decided to cluster these groups due to the small number of clinical studies available to evaluate each one of these cell populations separately. ILs different from IL-2 used for CD19 CAR T cell manufacturing showed higher OS rates despite similar BCR and lower BOR, evidencing the necessity of running clinical trials using different cytokines for CAR T cell expansion to evaluate their impacts on clinical responses. The CAR T cell activation and expansion methods were equivalent for all outcomes evaluated.

Considering the covariate age, patients under 18 had notably higher BCR and BOR rates but similar OS compared to older patients. CD19 CAR T cell therapy is known to induce a high clinical response rate in children and young adults, especially with B-ALL, but relapses are still a current issue [62], explaining, at least in part, the similar OS despite the higher BCR rates found in younger patients.

Regarding the CAR T cell dose effect, higher BCR, BOR, and OS rates were found for patients treated with doses between 1 and 4.9 million cells/kg compared to those with doses greater than 100 million cells/kg. The dose-effect might be biased considering the higher BCR and BOR rates found for younger patients, usually treated with lower CAR T cell doses. Nevertheless, the age bias can be ruled out for the higher OS rates observed for lower CAR T doses since OS was not affected by age. When the number of CAR T infusions was evaluated, we noted that three or more infusions presented lower rates for the evaluated outcomes. This result is critical because higher CAR T doses with repeated infusions are known to enhance toxicity [71, 72] despite the evident increased manufacturing cost. These results might affect the design of future comparative CD19 CAR T cells-based clinical trials, which can be focused on testing different dose scales up to 100 million cells/kg, limiting the administration to one or two infusions.

The comparison of different molecules used to build the structural CD19-directed CAR hinge (CD8, CD28, or IgG4), transmembrane (CD8 or CD28), and costimulatory domains (CD28 or 4-1BB) showed that the presence of CD28 in these three domains revealed higher rates for all the clinical outcomes evaluated. It might be possible that the different CAR domains act synergistically since they are part of the same functional full costimulatory molecule in human immune cells. However, we cannot affirm or discard this hypothesis based on our data. Particularly considering OS, the most relevant rate difference was found when CD28 was in the CAR’s hinge and costimulatory regions. For BCR, the rate differences between CD28 and other molecules tested were less relevant. A CAR hinge and transmembrane-based comparison with clinical data has never been performed before in the literature, and our meta-analysis gives us some evidence that must be further investigated in future studies to clarify the possibility of synergism combining different/ equal domains. For the costimulatory domains, data recently published in a meta-analysis focused on patients with diffuse large B-cell lymphoma (DLBCL) treated with CD19 CAR T cells corroborated our findings, showing higher BCR and BOR rates of CD28 (57% BCR and 81% BOR) compared to 4-1BB (42% BCR and 70% BOR). However, they found a non-significant statistical difference between CD28 and 4-1BB considering the 12-month OS rate for DLBCL patients [69].In the same study, the CD28-based Axi-cel had higher rates for all outcomes evaluated compared with the 4-1BB Tisa-cel CAR T for the treatment of DLBCL patients, with a BCR rate of 57% versus 36%, OS rate of 65% versus 49%, and BOR rate of 82% versus 58%, respectively [69]A clinical trial comparing CD19 CAR-T containing either CD28 or 4-1BB was performed to treat ten ALL patients, five treated with each type of construction in a dose of 0.62 × 10^6^ CAR T cells/kg. This study showed similar responses for both treatments, with the CD28 group resulting in 3 CR, 1 PR, and one no response (NR), and the 4-1BB with 3 CR, 0 PR, and 2 NR. Despite the superior number of NR patients in the 4-1BB group, this group had a unique patient with an ongoing anti-tumor response evaluated five months after treatment [73].This clinical trial was not conclusive due to the limited number of patients. Still considering the costimulatory domain of the CAR, Cappel and Kochenderfer recently reviewed and compared CAR T cell clinical studies based on different targets and having CD28 or 4-1BB as costimulatory domains, including but not limiting CD19 as a target. This general review showed that the available data from clinical trials do not demonstrate a clear advantage of either CD28-costimulated or 4-1BB-costimulated CARs for treating B cell lymphomas or B-ALL, pointing out that more extensive studies and comparative clinical trials must be performed to allow a conclusion about the performance of the different costimulatory domains against B-cell malignancies [74].

This study is the pioneer in evaluating the impact of the hinge and TMD CAR domains in addition to costimulatory domains in CD19 CAR T cell’s clinical response for B cell leukemia and lymphoma, which is an essential unanswered question in the field. In summary, several covariates analyzed might have a positive impact on all the evaluated clinical outcomes BCR, OS, and BOR of patients treated with CD19 CAR T cell therapies, such as age inferior to 18 years old, injection of 1 to 4.9 million CAR T cells per kg, with one CAR T cell infusion – without discard a potential efficiency using two doses – and CD28 constituting the hinge, transmembrane, and costimulatory domains of the CAR, as in Axi-cel product, and must be better explored in future comparative clinical trials.

The lack of randomized trials or large observational studies on CAR T cells justifies the implementation of this meta-analysis, which intends to provide insights on the ongoing procedures for further research, raising questions and spotting potential aspects of interest in the current approaches. Due to the unavoidable heterogeneity observed, the results of this meta-analysis are not deemed for clinical decision-making but to improve the understanding of this complex and multifaceted treatment instead. The extrapolation and generalization of the results obtained in this meta-analysis should be made with caution since it may be biased by the different study designs and characteristics considering CAR structures, CAR T cell manufacture conditions, doses, tumor type, autologous cells isolated from each individual heavily pretreated, and other variables.

### Electronic supplementary material

Below is the link to the electronic supplementary material.

Supplementary Material 1

## Data Availability

All data generated or analyzed during this study are included in this published article and its supplementary information files.

## Abbreviations

ALL: Acute lymphocytic leukemia
BCR: Best complete response
BOR: Best objective response
CAR: Chimeric antigen receptors
CLL: Chronic lymphocytic leukemia
CR: Complete response
DLBCL: Diffuse large B-cell lymphoma
HL: Hodgkin lymphoma
NHL: Non-Hodgkin lymphoma
OS: Overall survival
OR: Objective response
PBMC: Peripheral blood mononuclear cells
scFv: Single-chain variable fragment
TMD: Transmembrane domains
