# Supplementary material for: CD19 CAR T cells for B cell malignancies: a systematic review and meta-analysis focused on clinical impacts of CAR structural domains, manufacturing conditions, cellular product, doses, patient’s age, and tumor types
Source: BMC Cancer. 2024 Aug 22;24:1037. doi: 10.1186/s12885-024-12651-6 (PMC11340198; doi:10.1186/s12885-024-12651-6)
Supplement: Supplementary file 1 — Supplementary Material 1 [file 12885_2024_12651_MOESM1_ESM.docx]

**Supplementary Methods**

**Data syntheses**

A meta-analysis of single proportions was performed using the risk ratio as a summary measure for BCR, OS and BOR and the inverse variance method for pooling estimates. The logit transformation of the overall proportion was performed to allow a more intuitive data interpretation and was presented with confidence intervals of 95% (Page, McKenzie et al. 2021). Due to the expected heterogeneity across studies, a Random-Effects model was fitted. The Hartung-Knapp method was used to adjust the conﬁdence interval for the summary effect due to the expected variance for observational studies(Hartung and Knapp 2001). The Clopper-Pearson interval was used to calculate confidence intervals 95% (CI) for individual studies(Agresti and Coull 1998). The restricted maximum-likelihood estimator (RMLE) was used for the between-study variance (τ2) calculation, presented with CI(Veroniki, Jackson et al. 2016).

For BCR and BOR, it was considered the number of patients analyzed after the treatment and for OS, it was considered the number of patients that received the treatment. Heterogeneity was estimated with the I2 statistic(Higgins and Thompson 2002), with estimates between 50% and 75% considered moderate heterogeneity and greater than 75% as high heterogeneity. The Egger test and the Baujat plot were used to detect sources of heterogeneity (Sterne and Egger 2001, Baujat, Mahe et al. 2002). Publication bias was analyzed through funnel plots(Sterne and Egger 2001). Funnel asymmetry was tested through linear regression with p<0.05 for statistical significance(Balduzzi, Rucker et al. 2019).

The metaregression was performed with a limited set of categorical moderators that are common for all studies, namely (a) patient age, (b) Anti CD19 CAR type (CAR Name), (c) type of CAR costimulatory domain (costimulation), (d) Tumor type, and (e) study quality. We adopted a Mixed-Effects Model, the Restricted Maximum-Likelihood Estimator for the τ2 estimation, and the Knapp-Hartung method to calculate CI and p values to attenuate the false positive rate. The meta-regression results are regression coefficients with 95% confidence intervals (CIs)(Balduzzi, Rucker et al. 2019).

Since subgroup analysis relies on the hypothesis that studies are not carried out in single populations, it is assumed that each subgroup will present an overall effect, especially when are included variables with fixed levels such as age group, CAR T cell structure, and therapeutical protocol(Harrer 2022). Also, a common effect model was fitted as an alternate referential for effect comparison on the origin of potential differences in the observed effects and a mixed-effects model for subgroup analysis(Harrer 2022).

Given the challenges of performing large studies with CAR T cells and the difficulties involving restrictive inclusion criteria for patients, we decided to perform a comprehensive sensitivity analysis to provide insights regarding the potential moderators of this therapeutical approach, considering the wide variety of possible combinations in CAR T cell construction and applications. Thus, we produced forest plots with subgroups considering all the covariates mentioned above for primary and secondary outcomes. Despite the rule of thumb that analysis must be restricted to the main study question under the risk of spurious and misleading findings (Harrer, Apolinario-Hagen et al. 2019), the decision made lies on the fact that little is known about the effect sizes and direction of effects when so many approaches are compared. Moreover, a single-arm meta-analysis of proportions is a valuable tool to indicate possible directions for future research questions if taken carefully. For this reason, we maintained the core analyses in the main text and highlighted that the sensitivity analyses are subsidiary and are deemed to be considered for further studies.

Hence, the metaregression was performed with a limited set of categorical moderators that are common for all studies, namely (a) patient age, (b) Anti CD19 CAR type (CAR Name), (c) type of CAR costimulatory domain (costimulation), (d) Tumor type, and (e) study quality. We adopted a Mixed-Effects Model, the Restricted Maximum-Likelihood Estimator for the τ^2^ estimation, and the Knapp-Hartung method to calculate CI and *p* values to attenuate the false positive rate. The meta-regression results are regression coefficients with 95% confidence intervals (CIs)(Shadish, Hedges et al. 2014).

For the risk of bias, certainty assessment is reported as 95% CI without a previous definition of the limits considering that there are no established settings of clinical practice guidelines.

**Supplementary Figures**





Suppl. Figure 1. Forest Plot representing the overall proportion of the secondary outcome 12-months overall survival (OS) of patients treated with anti-CD19 CAR T therapy based on the studies included in the meta-analysis.





Suppl. Figure 2. Forest Plot representing the overall proportion of the secondary outcome Best Objective Response (BOR) of patients treated with anti-CD19 CAR T therapy based on the studies included in the meta-analysis.


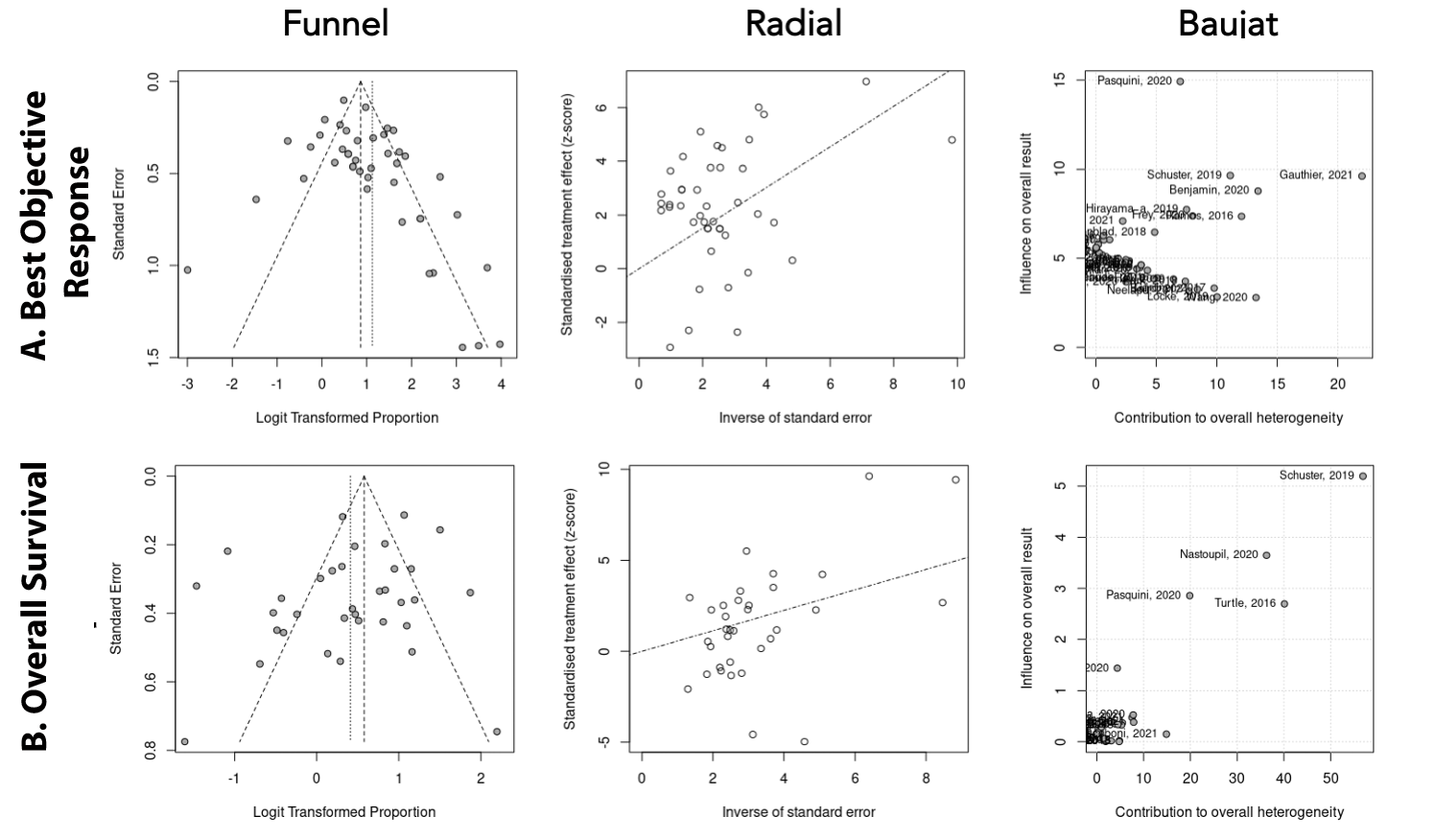


Suppl. Figure 3. Funnel, Baujat, and Radial plots showing the heterogeneity observed for the secondary outcomes **A.** Best Objective Response (BOR) and **B.** 12-months overall survival (OS) of patients treated with anti-CD19 CAR T therapy based on the studies included in this systematic review and meta-analysis.





Suppl. Figure 4. Forest Plot representing the proportion of the primary outcome Best Complete Response (BCR) stratified according to the covariable age (class inferior or superior to 18 years old) of patients treated with anti-CD19 CAR T therapy based on the studies included in the meta-analysis.





Suppl. Figure 5. Forest Plot representing the proportion of the secondary outcome 12-months overall survival (OS) stratified according to the covariable age (class inferior or superior to 18 years old) of patients treated with anti-CD19 CAR T therapy based on the studies included in the meta-analysis.





Suppl. Figure 6. Forest Plot representing the proportion of the secondary outcome Best Objective Response (BOR) stratified according to the covariable age (class inferior or superior to 18 years old) of patients treated with anti-CD19 CAR T therapy based on the studies included in the meta-analysis.





Suppl. Figure 7. Forest Plot representing the proportion of the primary outcome Best Complete Response (BCR) stratified according to the covariable interleukin (IL) used to cultivate CAR T cells in vitro (IL-2; IL-2+other; IL-7+IL-15; or others) of patients treated with anti-CD19 CAR T therapy based on the studies included in the meta-analysis.





Suppl. Figure 8. Forest Plot representing the proportion of the secondary outcome 12-months overall survival (OS) stratified according to the covariable interleukin (IL) used to cultivate CAR T cells in vitro (IL-2; IL-2+other; IL-7+IL-15; or others) of patients treated with anti-CD19 CAR T therapy based on the studies included in the meta-analysis.





Suppl. Figure 9. Forest Plot representing the proportion of the secondary outcome Best Objective Response (BOR) stratified according to the covariable interleukin (IL) used to cultivate CAR T cells in vitro (IL-2; IL-2+other; IL-7+IL-15; or others) of patients treated with anti-CD19 CAR T therapy based on the studies included in the meta-analysis.





Suppl. Figure 10. Forest Plot representing the proportion of the primary outcome Best Complete Response (BCR) stratified according to the covariable method to activate CAR T cells in vitro (Anti-CD3 monoclonal antibodies – mAb, Anti-CD3/ Anti-CD28 Beads, or Irradiated feeder cells) of patients treated with anti-CD19 CAR T therapy based on the studies included in the meta-analysis.





Suppl. Figure 11. Forest Plot representing the proportion of the secondary outcome 12-months overall survival (OS) stratified according to the covariable method to activate CAR T cells in vitro (Anti-CD3 monoclonal antibodies – mAb, Anti-CD3/ Anti-CD28 Beads, or Irradiated feeder cells) of patients treated with anti-CD19 CAR T therapy based on the studies included in the meta-analysis.





Suppl. Figure 12. Forest Plot representing the proportion of the secondary outcome Best Objective Response (BOR) stratified according to the covariable method to activate CAR T cells in vitro (Anti-CD3 monoclonal antibodies – mAb, Anti-CD3/ Anti-CD28 Beads, or Irradiated feeder cells) of patients treated with anti-CD19 CAR T therapy based on the studies included in the meta-analysis.





Suppl. Figure 13. Forest Plot representing the proportion of the primary outcome Best Complete Response (BCR) stratified according to the covariable cell type to produce the CAR T (PBMCs or other specific subsets) of patients treated with anti-CD19 CAR T therapy based on the studies included in the meta-analysis.





Suppl. Figure 14. Forest Plot representing the proportion of the secondary outcome Best Objective Response (BOR) stratified according to the covariable cell type to produce the CAR T (PBMCs or other specific subsets) of patients treated with anti-CD19 CAR T therapy based on the studies included in the meta-analysis.





Suppl. Figure 15. Forest Plot representing the proportion of the secondary outcome 12-months overall survival (OS) stratified according to the covariable cell type to produce the CAR T (PBMCs or other specific subsets) of patients treated with anti-CD19 CAR T therapy based on the studies included in the meta-analysis.





Suppl. Figure 16. Forest Plot representing the proportion of the primary outcome Best Complete Response (BCR) stratified according to the covariable number of injected cells/Kg (≤9.9x10e5; 1 to 4.9x10e6; 5x10e6 to 9.9x10e7; ≥10e8; variable cell numbers) of patients treated with anti-CD19 CAR T therapy based on the studies included in the meta-analysis.





Suppl. Figure 17. Forest Plot representing the proportion of the secondary outcome 12-months overall survival (OS) stratified according to the covariable number of injected cells/Kg (≤9.9x10e5; 1 to 4.9x10e6; 5x10e6 to 9.9x10e7; ≥10e8; variable cell numbers) of patients treated with anti-CD19 CAR T therapy based on the studies included in the meta-analysis.





Suppl. Figure 18. Forest Plot representing the proportion of the secondary outcome Best Objective Response (BOR) stratified according to the covariable number of injected cells/Kg (≤9.9x10e5; 1 to 4.9x10e6; 5x10e6 to 9.9x10e7; ≥10e8; variable cell numbers) of patients treated with anti-CD19 CAR T therapy based on the studies included in the meta-analysis.





Suppl. Figure 19. Forest Plot representing the proportion of the primary outcome Best Complete Response (BCR) stratified according to the covariable number of CAR T infusions (one, two, ≥3 or variable) of patients treated with anti-CD19 CAR T therapy based on the studies included in the meta-analysis.





Suppl. Figure 20. Forest Plot representing the proportion of the secondary outcome 12-months overall survival (OS) stratified according to the covariable number of CAR T infusions (one, two, ≥3 or variable) of patients treated with anti-CD19 CAR T therapy based on the studies included in the meta-analysis.





Suppl. Figure 21. Forest Plot representing the proportion of the secondary outcome Best Objective Response (BOR) stratified according to the covariable number of CAR T infusions (one, two, ≥3 or variable) of patients treated with anti-CD19 CAR T therapy based on the studies included in the meta-analysis.





Suppl. Figure 22. Forest Plot representing the proportion of the primary outcome Best Complete Response (BCR) stratified according to the covariable CAR T name (Axi-cel, Tisa-cel, JCAR014, MDACC, others) of patients treated with anti-CD19 CAR T therapy based on the studies included in the meta-analysis.





Suppl. Figure 23. Forest Plot representing the proportion of the secondary outcome 12-months overall survival (OS) stratified according to the covariable CAR T name (Axi-cel, Tisa-cel, JCAR014, MDACC, others) of patients treated with anti-CD19 CAR T therapy based on the studies included in the meta-analysis.





Suppl. Figure 24. Forest Plot representing the proportion of the secondary outcome Best Objective Response (BOR) stratified according to the covariable CAR T name (Axi-cel, Tisa-cel, JCAR014, MDACC, others) of patients treated with anti-CD19 CAR T therapy based on the studies included in the meta-analysis.





Suppl. Figure 25. Forest Plot representing the proportion of the primary outcome Best Complete Response (BCR) stratified according to the covariable CAR hinge domain (CD8, CD28, IgG1 or IgG4) of patients treated with anti-CD19 CAR T therapy based on the studies included in the meta-analysis.





Suppl. Figure 26. Forest Plot representing the proportion of the secondary outcome 12-months overall survival (OS) stratified according to the covariable CAR hinge domain (CD8, CD28, IgG1 or IgG4) of patients treated with anti-CD19 CAR T therapy based on the studies included in the meta-analysis.





Suppl. Figure 27. Forest Plot representing the proportion of the secondary outcome Best Objective Response (BOR) stratified according to the covariable CAR hinge domain (CD8, CD28, IgG1 or IgG4) of patients treated with anti-CD19 CAR T therapy based on the studies included in the meta-analysis.





Suppl. Figure 28. Forest Plot representing the proportion of the primary outcome Best Complete Response (BCR) stratified according to the covariable CAR transmembrane domain (CD8, CD28, other/mixed) of patients treated with anti-CD19 CAR T therapy based on the studies included in the meta-analysis.





Suppl. Figure 29. Forest Plot representing the proportion of the secondary outcome 12-months overall survival (OS) stratified according to the covariable CAR transmembrane domain (CD8, CD28, other/mixed) of patients treated with anti-CD19 CAR T therapy based on the studies included in the meta-analysis.





Suppl. Figure 30. Forest Plot representing the proportion of the secondary outcome Best Objective Response (BOR) stratified according to the covariable CAR transmembrane domain (CD8, CD28, other/mixed) of patients treated with anti-CD19 CAR T therapy based on the studies included in the meta-analysis.





Suppl. Figure 31. Forest Plot representing the proportion of the primary outcome Best Complete Response (BCR) stratified according to the covariable CAR T costimulation (1^st^ generation, 2^nd^ generation CD28, 2^nd^ generation 4-1BB, 3^rd^ generation CD28/4-1BB, 4^th^ generation CD28/4-1BB) of patients treated with anti-CD19 CAR T therapy based on the studies included in the meta-analysis.





Suppl. Figure 32. Forest Plot representing the proportion of the secondary outcome 12-months overall survival (OS) stratified according to the covariable CAR T costimulation (1^st^ generation, 2^nd^ generation CD28, 2^nd^ generation 4-1BB, 3^rd^ generation CD28/4-1BB, 4^th^ generation CD28/4-1BB) of patients treated with anti-CD19 CAR T therapy based on the studies included in the meta-analysis.





Suppl. Figure 33. Forest Plot representing the proportion of the secondary outcome Best Objective Response (BOR) stratified according to the covariable CAR T costimulation (1^st^ generation, 2^nd^ generation CD28, 2^nd^ generation 4-1BB, 3^rd^ generation CD28/4-1BB, 4^th^ generation CD28/4-1BB) of patients treated with anti-CD19 CAR T therapy based on the studies included in the meta-analysis.





Suppl. Figure 34. Forest Plot representing the proportion of the primary outcome Best Complete Response (BCR) stratified according to the covariable tumor type [acute lymphocytic leukemia (ALL); chronic lymphocytic leukemia (CLL); non-Hodgkin lymphoma (NHL); NHL+Hodgkin lymphoma (HL); CLL+ALL+NHL; NHL+ALL; NHL+CLL] of patients treated with anti-CD19 CAR T therapy based on the studies included in the meta-analysis.





Suppl. Figure 35. Forest Plot representing the proportion of the secondary outcome 12-months overall survival (OS) stratified according to the covariable tumor type [acute lymphocytic leukemia (ALL); chronic lymphocytic leukemia (CLL); non-Hodgkin lymphoma (NHL); NHL+Hodgkin lymphoma (HL); CLL+ALL+NHL; NHL+ALL; NHL+CLL] of patients treated with anti-CD19 CAR T therapy based on the studies included in the meta-analysis.





Suppl. Figure 36. Forest Plot representing the proportion of the secondary outcome Best Objective Response (BOR) stratified according to the covariable tumor type [acute lymphocytic leukemia (ALL); chronic lymphocytic leukemia (CLL); non-Hodgkin lymphoma (NHL); NHL+Hodgkin lymphoma (HL); CLL+ALL+NHL; NHL+ALL; NHL+CLL] of patients treated with anti-CD19 CAR T therapy based on the studies included in the meta-analysis.





Suppl. Figure 37. Forest Plot representing the proportion of the primary outcome Best Complete Response (BCR) stratified according to the risk of bias of patients treated with anti-CD19 CAR T therapy based on the studies included in the meta-analysis. The risk of bias was accessed as described in Methods.





Suppl. Figure 38. Forest Plot representing the proportion of the secondary outcome 12-months overall survival (OS) stratified according to the risk of bias of patients treated with anti-CD19 CAR T therapy based on the studies included in the meta-analysis. The risk of bias was accessed as described in Methods.





Suppl. Figure 39. Forest Plot representing the proportion of the secondary outcome Best Objective Response (BOR) stratified according to the risk of bias of patients treated with anti-CD19 CAR T therapy based on the studies included in the meta-analysis. The risk of bias was accessed as described in Methods.

**Suppl. Table 1.** Best Complete Response subgroup analysis

| **Variable** | **Proportion per subgroup** | **95% CI** | **I^2^ (%)** | **References** |
| --- | --- | --- | --- | --- |
| **General** | 0.56 | 0.49 – 0.63 | 81 | (Kochenderfer, Dudley et al. 2015, Lee, Kochenderfer et al. 2015, Bhoj, Arhontoulis et al. 2016, Kebriaei, Singh et al. 2016, Ramos, Savoldo et al. 2016, Turtle, Hanafi et al. 2016, Gardner, Finney et al. 2017, Hu, Wu et al. 2017, Neelapu, Locke et al. 2017, Schuster, Svoboda et al. 2017, Turtle, Hay et al. 2017, Enblad, Karlsson et al. 2018, Jacoby, Bielorai et al. 2018, Maude, Laetsch et al. 2018, Park, Rivière et al. 2018, Rossi, Paczkowski et al. 2018, Curran, Margossian et al. 2019, Ghorashian, Kramer et al. 2019, Hay, Gauthier et al. 2019, Hirayama, Gauthier et al. 2019, Hirayama, Gauthier et al. 2019, Locke, Ghobadi et al. 2019, Schuster, Bishop et al. 2019, Ying, Huang et al. 2019, Abramson, Palomba et al. 2020, An, Wang et al. 2020, Benjamin, Graham et al. 2020, Cappell, Sherry et al. 2020, Frey, Gill et al. 2020, Gu, Liu et al. 2020, Jacobson, Hunter et al. 2020, Liu, Marin et al. 2020, Nastoupil, Jain et al. 2020, Pasquini, Hu et al. 2020, Sesques, Ferrant et al. 2020, Wang, Jiang et al. 2020, Zhou, Tu et al. 2020, Baird, Epstein et al. 2021, Gauthier, Bezerra et al. 2021, Iacoboni, Villacampa et al. 2021, Mian, Wei et al. 2021, Shah, Bishop et al. 2021, Tan, Pan et al. 2021, Wang, Wang et al. 2021, Ying, Yang et al. 2021) |
| **Age** | | | | |
| <18 | 0.79 | 0.65-0.89 | 64 | (Lee, Kochenderfer et al. 2015, Gardner, Finney et al. 2017, Jacoby, Bielorai et al. 2018, Maude, Laetsch et al. 2018, Curran, Margossian et al. 2019, Ghorashian, Kramer et al. 2019, Tan, Pan et al. 2021, Wang, Wang et al. 2021) |
| >18 | 0.51 | 0.43-0.57 | 82 | (Kochenderfer, Dudley et al. 2015, Bhoj, Arhontoulis et al. 2016, Kebriaei, Singh et al. 2016, Ramos, Savoldo et al. 2016, Turtle, Hanafi et al. 2016, Hu, Wu et al. 2017, Neelapu, Locke et al. 2017, Schuster, Svoboda et al. 2017, Turtle, Hay et al. 2017, Enblad, Karlsson et al. 2018, Park, Rivière et al. 2018, Rossi, Paczkowski et al. 2018, Hay, Gauthier et al. 2019, Hirayama, Gauthier et al. 2019, Hirayama, Gauthier et al. 2019, Locke, Ghobadi et al. 2019, Schuster, Bishop et al. 2019, Ying, Huang et al. 2019, Abramson, Palomba et al. 2020, An, Wang et al. 2020, Benjamin, Graham et al. 2020, Cappell, Sherry et al. 2020, Chen, Zhang et al. 2020, Frey, Gill et al. 2020, Gu, Liu et al. 2020, Jacobson, Hunter et al. 2020, Liu, Marin et al. 2020, Nastoupil, Jain et al. 2020, Pasquini, Hu et al. 2020, Sesques, Ferrant et al. 2020, Wang, Munoz et al. 2020, Zhou, Tu et al. 2020, Baird, Epstein et al. 2021, Gauthier, Bezerra et al. 2021, Iacoboni, Villacampa et al. 2021, Mian, Wei et al. 2021, Shah, Bishop et al. 2021, Ying, Yang et al. 2021) |
| **Interleukin used for**  **CAR T cell expansion** | | | | |
| IL-2 | 0.58 | 0.50-0.66 | 76 | (Kochenderfer, Dudley et al. 2015, Lee, Kochenderfer et al. 2015, Turtle, Hanafi et al. 2016, Hu, Wu et al. 2017, Neelapu, Locke et al. 2017, Turtle, Hay et al. 2017, Enblad, Karlsson et al. 2018, Jacoby, Bielorai et al. 2018, Rossi, Paczkowski et al. 2018, Hay, Gauthier et al. 2019, Hirayama, Gauthier et al. 2019, Hirayama, Gauthier et al. 2019, Locke, Ghobadi et al. 2019, Ying, Huang et al. 2019, An, Wang et al. 2020, Benjamin, Graham et al. 2020, Cappell, Sherry et al. 2020, Chen, Zhang et al. 2020, Gu, Liu et al. 2020, Jacobson, Hunter et al. 2020, Liu, Marin et al. 2020, Nastoupil, Jain et al. 2020, Sesques, Ferrant et al. 2020, Wang, Munoz et al. 2020, Baird, Epstein et al. 2021, Mian, Wei et al. 2021, Shah, Bishop et al. 2021, Tan, Pan et al. 2021) |
| Others/ Not mentioned | 0.54 | 0.43-0.65 | 72 | (Bhoj, Arhontoulis et al. 2016, Schuster, Svoboda et al. 2017, Maude, Laetsch et al. 2018, Park, Rivière et al. 2018, Curran, Margossian et al. 2019, Ghorashian, Kramer et al. 2019, Schuster, Bishop et al. 2019, Abramson, Palomba et al. 2020, Pasquini, Hu et al. 2020, Zhou, Tu et al. 2020, Iacoboni, Villacampa et al. 2021) |
| **CAR T Cells Activation**  **Method** | | | | |
| Anti-CD3 mAb | 0.55 | 0.43 – 0.67 | 71 | (Kochenderfer, Dudley et al. 2015, Lee, Kochenderfer et al. 2015, Kebriaei, Singh et al. 2016, Ramos, Savoldo et al. 2016, Enblad, Karlsson et al. 2018, Jacoby, Bielorai et al. 2018, Locke, Ghobadi et al. 2019, Schuster, Bishop et al. 2019, Cappell, Sherry et al. 2020, Sesques, Ferrant et al. 2020, Wang, Munoz et al. 2020, Shah, Bishop et al. 2021, Wang, Wang et al. 2021) |
| Anti-CD3/CD28 beads | 0.56 | 0.47 – 0.65 | 84 | (Bhoj, Arhontoulis et al. 2016, Turtle, Hanafi et al. 2016, Gardner, Finney et al. 2017, Hu, Wu et al. 2017, Neelapu, Locke et al. 2017, Schuster, Svoboda et al. 2017, Turtle, Hay et al. 2017, Maude, Laetsch et al. 2018, Park, Rivière et al. 2018, Rossi, Paczkowski et al. 2018, Curran, Margossian et al. 2019, Ghorashian, Kramer et al. 2019, Hay, Gauthier et al. 2019, Hirayama, Gauthier et al. 2019, Hirayama, Gauthier et al. 2019, Ying, Huang et al. 2019, Abramson, Palomba et al. 2020, An, Wang et al. 2020, Benjamin, Graham et al. 2020, Frey, Gill et al. 2020, Gu, Liu et al. 2020, Jacobson, Hunter et al. 2020, Nastoupil, Jain et al. 2020, Pasquini, Hu et al. 2020, Sesques, Ferrant et al. 2020, Zhou, Tu et al. 2020, Baird, Epstein et al. 2021, Gauthier, Bezerra et al. 2021, Iacoboni, Villacampa et al. 2021, Mian, Wei et al. 2021, Tan, Pan et al. 2021, Ying, Yang et al. 2021) |
| **Number CAR T cells**  **injected/ kg** | | | | |
| 1 to 4,9x10e6 | 0.63 | 0.55-0.71 | 77 | (Lee, Kochenderfer et al. 2015, Bhoj, Arhontoulis et al. 2016, Turtle, Hanafi et al. 2016, Gardner, Finney et al. 2017, Hu, Wu et al. 2017, Neelapu, Locke et al. 2017, Turtle, Hay et al. 2017, Jacoby, Bielorai et al. 2018, Maude, Laetsch et al. 2018, Park, Rivière et al. 2018, Curran, Margossian et al. 2019, Ghorashian, Kramer et al. 2019, Hay, Gauthier et al. 2019, Hirayama, Gauthier et al. 2019, Hirayama, Gauthier et al. 2019, Locke, Ghobadi et al. 2019, An, Wang et al. 2020, Cappell, Sherry et al. 2020, Jacobson, Hunter et al. 2020, Liu, Marin et al. 2020, Nastoupil, Jain et al. 2020, Sesques, Ferrant et al. 2020, Wang, Munoz et al. 2020, Baird, Epstein et al. 2021, Gauthier, Bezerra et al. 2021, Mian, Wei et al. 2021, Shah, Bishop et al. 2021, Tan, Pan et al. 2021, Wang, Wang et al. 2021) |
| 5x10e6 to 9,9x10e7 | 0.71 | 0.25-0.95 | 62 | (Kochenderfer, Dudley et al. 2015, Schuster, Svoboda et al. 2017, Gu, Liu et al. 2020) |
| ≥10e8 | 0.36 | 0.28-0.46 | 38 | (Kebriaei, Singh et al. 2016, Ramos, Savoldo et al. 2016, Enblad, Karlsson et al. 2018, Schuster, Bishop et al. 2019, Ying, Huang et al. 2019, Iacoboni, Villacampa et al. 2021, Ying, Yang et al. 2021) |
| Variable | 0.40 | 0.25-0.56 | 80 | (Rossi, Paczkowski et al. 2018, Abramson, Palomba et al. 2020, Benjamin, Graham et al. 2020, Frey, Gill et al. 2020, Pasquini, Hu et al. 2020) |
| **Number of CAR T cells**  **infusions** | | | | |
| One infusion | 0.55 | 0.48-0.62 | 81 | (Kochenderfer, Dudley et al. 2015, Kebriaei, Singh et al. 2016, Ramos, Savoldo et al. 2016, Turtle, Hanafi et al. 2016, Neelapu, Locke et al. 2017, Schuster, Svoboda et al. 2017, Turtle, Hay et al. 2017, Enblad, Karlsson et al. 2018, Jacoby, Bielorai et al. 2018, Maude, Laetsch et al. 2018, Ghorashian, Kramer et al. 2019, Hirayama, Gauthier et al. 2019, Hirayama, Gauthier et al. 2019, Locke, Ghobadi et al. 2019, Schuster, Bishop et al. 2019, An, Wang et al. 2020, Cappell, Sherry et al. 2020, Gu, Liu et al. 2020, Jacobson, Hunter et al. 2020, Nastoupil, Jain et al. 2020, Pasquini, Hu et al. 2020, Sesques, Ferrant et al. 2020, Wang, Munoz et al. 2020, Zhou, Tu et al. 2020, Baird, Epstein et al. 2021, Iacoboni, Villacampa et al. 2021, Mian, Wei et al. 2021, Shah, Bishop et al. 2021, Tan, Pan et al. 2021, Wang, Wang et al. 2021, Ying, Yang et al. 2021) |
| Two infusions | 0.65 | 0.00-1.0 | 97 | (Gardner, Finney et al. 2017, Gauthier, Bezerra et al. 2021) |
| ≥3 infusions | 0.50 | 0.25-0.74 | 74 | (Hu, Wu et al. 2017, Park, Rivière et al. 2018, Curran, Margossian et al. 2019, Frey, Gill et al. 2020) |
| Variable | 0.61 | 0.33-0.83 | 79 | (Liu, Smyth et al. , Lee, Kochenderfer et al. 2015, Bhoj, Arhontoulis et al. 2016, Rossi, Paczkowski et al. 2018, Hay, Gauthier et al. 2019, Ying, Huang et al. 2019, Abramson, Palomba et al. 2020, Benjamin, Graham et al. 2020, Liu, Marin et al. 2020) |
| **Cell population transduced**  **or transfected with the CAR** | | | | |
| PBMCs | 0.57 | 0.49 – 0.64 | 80 | (Kochenderfer, Dudley et al. 2015, Lee, Kochenderfer et al. 2015, Bhoj, Arhontoulis et al. 2016, Kebriaei, Singh et al. 2016, Ramos, Savoldo et al. 2016, Hu, Wu et al. 2017, Neelapu, Locke et al. 2017, Schuster, Svoboda et al. 2017, Enblad, Karlsson et al. 2018, Jacoby, Bielorai et al. 2018, Maude, Laetsch et al. 2018, Park, Rivière et al. 2018, Rossi, Paczkowski et al. 2018, Curran, Margossian et al. 2019, Ghorashian, Kramer et al. 2019, Locke, Ghobadi et al. 2019, Schuster, Bishop et al. 2019, Ying, Huang et al. 2019, An, Wang et al. 2020, Benjamin, Graham et al. 2020, Cappell, Sherry et al. 2020, Chen, Zhang et al. 2020, Frey, Gill et al. 2020, Gu, Liu et al. 2020, Jacobson, Hunter et al. 2020, Liu, Marin et al. 2020, Nastoupil, Jain et al. 2020, Pasquini, Hu et al. 2020, Sesques, Ferrant et al. 2020, Wang, Munoz et al. 2020, Zhou, Tu et al. 2020, Iacoboni, Villacampa et al. 2021, Mian, Wei et al. 2021, Shah, Bishop et al. 2021, Tan, Pan et al. 2021, Wang, Wang et al. 2021) |
| CD4/CD8 1:1 or CD8+ only or specific subsets | 0.54 | 0.34-0.73 | 87 | (Turtle, Hanafi et al. 2016, Gardner, Finney et al. 2017, Turtle, Hay et al. 2017, Hay, Gauthier et al. 2019, Hirayama, Gauthier et al. 2019, Hirayama, Gauthier et al. 2019, Abramson, Palomba et al. 2020, Baird, Epstein et al. 2021, Gauthier, Bezerra et al. 2021, Ying, Yang et al. 2021) |
| **Anti-CD19 CAR Type (Name)** | | | | |
| Axicabtagene citoleucel (Yescarta) (Axi-cel) KTE-C19 | 0.62 | 0.56 – 0.67 | 52 | (Neelapu, Locke et al. 2017, Jacoby, Bielorai et al. 2018, Park, Rivière et al. 2018, Rossi, Paczkowski et al. 2018, Locke, Ghobadi et al. 2019, Cappell, Sherry et al. 2020, Chen, Zhang et al. 2020, Jacobson, Hunter et al. 2020, Liu, Marin et al. 2020, Nastoupil, Jain et al. 2020, Sesques, Ferrant et al. 2020, Wang, Munoz et al. 2020, Baird, Epstein et al. 2021, Mian, Wei et al. 2021) |
| Tisagenlecleucel (CTL019) (Kymriah) (Tisa-cel) | 0.53 | 0.38-0.67 | 66 | (Kochenderfer, Dudley et al. 2015, Lee, Kochenderfer et al. 2015, Bhoj, Arhontoulis et al. 2016, Hu, Wu et al. 2017, Schuster, Svoboda et al. 2017, Ying, Huang et al. 2019, Frey, Gill et al. 2020, Pasquini, Hu et al. 2020, Sesques, Ferrant et al. 2020, Iacoboni, Villacampa et al. 2021, Tan, Pan et al. 2021) |
| JCAR014 | 0.44 | 0.20-0.71 | 88 | (Turtle, Hanafi et al. 2016, Turtle, Hay et al. 2017, Hay, Gauthier et al. 2019, Hirayama, Gauthier et al. 2019, Hirayama, Gauthier et al. 2019, Gauthier, Bezerra et al. 2021) |
| Others | 0.60 | 0.40-0.78 | 82 | (Ramos, Savoldo et al. 2016, Gardner, Finney et al. 2017, Enblad, Karlsson et al. 2018, Ghorashian, Kramer et al. 2019, Abramson, Palomba et al. 2020, An, Wang et al. 2020, Benjamin, Graham et al. 2020, Gu, Liu et al. 2020, Zhou, Tu et al. 2020, Shah, Bishop et al. 2021, Wang, Wang et al. 2021, Ying, Yang et al. 2021) |
| **CAR Hinge**  **Domain** | | | | |
| CD8 | 0.56 | 0.42-0.70 | 75 | (Kochenderfer, Dudley et al. 2015, Lee, Kochenderfer et al. 2015, Bhoj, Arhontoulis et al. 2016, Hu, Wu et al. 2017, Schuster, Svoboda et al. 2017, Maude, Laetsch et al. 2018, Ghorashian, Kramer et al. 2019, Schuster, Bishop et al. 2019, Ying, Huang et al. 2019, Benjamin, Graham et al. 2020, Frey, Gill et al. 2020, Gu, Liu et al. 2020, Pasquini, Hu et al. 2020, Sesques, Ferrant et al. 2020, Zhou, Tu et al. 2020, Iacoboni, Villacampa et al. 2021, Tan, Pan et al. 2021, Wang, Wang et al. 2021) |
| CD28 | 0.60 | 0.55-0.66 | 52 | (Neelapu, Locke et al. 2017, Enblad, Karlsson et al. 2018, Jacoby, Bielorai et al. 2018, Park, Rivière et al. 2018, Rossi, Paczkowski et al. 2018, Locke, Ghobadi et al. 2019, Cappell, Sherry et al. 2020, Jacobson, Hunter et al. 2020, Liu, Marin et al. 2020, Nastoupil, Jain et al. 2020, Sesques, Ferrant et al. 2020, Wang, Munoz et al. 2020, Baird, Epstein et al. 2021, Mian, Wei et al. 2021, Shah, Bishop et al. 2021) |
| IgG4 | 0.50 | 0.35-0.66 | 85 | (Kebriaei, Singh et al. 2016, Turtle, Hanafi et al. 2016, Turtle, Hay et al. 2017, Curran, Margossian et al. 2019, Hay, Gauthier et al. 2019, Hirayama, Gauthier et al. 2019, Hirayama, Gauthier et al. 2019, Abramson, Palomba et al. 2020, An, Wang et al. 2020, Gauthier, Bezerra et al. 2021, Ying, Yang et al. 2021) |
| **CAR Transmembrane**  **Domain** | | | | |
| CD8 | 0.54 | 0.40-0.68 | 73 | (Kochenderfer, Dudley et al. 2015, Lee, Kochenderfer et al. 2015, Bhoj, Arhontoulis et al. 2016, Hu, Wu et al. 2017, Schuster, Svoboda et al. 2017, Maude, Laetsch et al. 2018, Ghorashian, Kramer et al. 2019, Schuster, Bishop et al. 2019, Ying, Huang et al. 2019, Benjamin, Graham et al. 2020, Frey, Gill et al. 2020, Pasquini, Hu et al. 2020, Sesques, Ferrant et al. 2020, Iacoboni, Villacampa et al. 2021, Tan, Pan et al. 2021, Wang, Wang et al. 2021) |
| CD28 | 0.58 | 0.48-0.67 | 80 | (Kebriaei, Singh et al. 2016, Ramos, Savoldo et al. 2016, Turtle, Hanafi et al. 2016, Gardner, Finney et al. 2017, Neelapu, Locke et al. 2017, Turtle, Hay et al. 2017, Enblad, Karlsson et al. 2018, Jacoby, Bielorai et al. 2018, Park, Rivière et al. 2018, Rossi, Paczkowski et al. 2018, Curran, Margossian et al. 2019, Hay, Gauthier et al. 2019, Hirayama, Gauthier et al. 2019, Hirayama, Gauthier et al. 2019, Locke, Ghobadi et al. 2019, An, Wang et al. 2020, Cappell, Sherry et al. 2020, Gu, Liu et al. 2020, Jacobson, Hunter et al. 2020, Liu, Marin et al. 2020, Nastoupil, Jain et al. 2020, Sesques, Ferrant et al. 2020, Wang, Munoz et al. 2020, Zhou, Tu et al. 2020, Baird, Epstein et al. 2021, Gauthier, Bezerra et al. 2021, Mian, Wei et al. 2021, Shah, Bishop et al. 2021) |
| **CAR Costimulatory**  **Domain** | | | | |
| CD28 | 0.60 | 0.54-0.66 | 55 | (Kebriaei, Singh et al. 2016, Neelapu, Locke et al. 2017, Jacoby, Bielorai et al. 2018, Park, Rivière et al. 2018, Rossi, Paczkowski et al. 2018, Curran, Margossian et al. 2019, Locke, Ghobadi et al. 2019, Cappell, Sherry et al. 2020, Jacobson, Hunter et al. 2020, Liu, Marin et al. 2020, Nastoupil, Jain et al. 2020, Sesques, Ferrant et al. 2020, Wang, Munoz et al. 2020, Baird, Epstein et al. 2021, Mian, Wei et al. 2021, Shah, Bishop et al. 2021) |
| 4-1BB | 0.56 | 0.44-0.67 | 82 | (Kochenderfer, Dudley et al. 2015, Lee, Kochenderfer et al. 2015, Bhoj, Arhontoulis et al. 2016, Turtle, Hanafi et al. 2016, Gardner, Finney et al. 2017, Hu, Wu et al. 2017, Schuster, Svoboda et al. 2017, Turtle, Hay et al. 2017, Maude, Laetsch et al. 2018, Ghorashian, Kramer et al. 2019, Hay, Gauthier et al. 2019, Hirayama, Gauthier et al. 2019, Hirayama, Gauthier et al. 2019, Schuster, Bishop et al. 2019, Ying, Huang et al. 2019, Abramson, Palomba et al. 2020, Benjamin, Graham et al. 2020, Chen, Zhang et al. 2020, Frey, Gill et al. 2020, Gu, Liu et al. 2020, Pasquini, Hu et al. 2020, Sesques, Ferrant et al. 2020, Gauthier, Bezerra et al. 2021, Iacoboni, Villacampa et al. 2021, Tan, Pan et al. 2021, Wang, Wang et al. 2021, Ying, Yang et al. 2021) |
| **Tumor Type** | | | | |
| NHL | 0.51 | 0.45-0.57 | 75 | (Turtle, Hanafi et al. 2016, Neelapu, Locke et al. 2017, Schuster, Svoboda et al. 2017, Rossi, Paczkowski et al. 2018, Svoboda, Rheingold et al. 2018, Hirayama, Gauthier et al. 2019, Hirayama, Gauthier et al. 2019, Locke, Ghobadi et al. 2019, Abramson, Palomba et al. 2020, Jacobson, Hunter et al. 2020, Nastoupil, Jain et al. 2020, Sesques, Ferrant et al. 2020, Zhou, Tu et al. 2020, Baird, Epstein et al. 2021, Iacoboni, Villacampa et al. 2021, Mian, Wei et al. 2021, Ying, Yang et al. 2021) |
| ALL | 0.73 | 0.60-0.83 | 77 | (Lee, Kochenderfer et al. 2015, Gardner, Finney et al. 2017, Hu, Wu et al. 2017, Jacoby, Bielorai et al. 2018, Maude, Laetsch et al. 2018, Park, Rivière et al. 2018, Curran, Margossian et al. 2019, Ghorashian, Kramer et al. 2019, Hay, Gauthier et al. 2019, An, Wang et al. 2020, Benjamin, Graham et al. 2020, Chen, Zhang et al. 2020, Gu, Liu et al. 2020, Shah, Bishop et al. 2021, Tan, Pan et al. 2021, Wang, Wang et al. 2021) |

**Suppl. Table 2.** Overall survival subgroup analysis

| Variable | Proportion per subgroup | 95% CI | I^2^ (%) | References |
| --- | --- | --- | --- | --- |
| General | 0.60 | 0.53-0.67 | 87 | (Abramson, Palomba et al. 2020); (Baird, Epstein et al. 2021); (Benjamin, Graham et al. 2020); (Cappell, Sherry et al. 2020, Chen, Zhang et al. 2020); (Curran, Margossian et al. 2019); (Enblad, Karlsson et al. 2018); (Frey, Gill et al. 2020) (Gardner, Finney et al. 2017); (Ghorashian, Kramer et al. 2019); (Gu, Liu et al. 2020); (Iacoboni, Villacampa et al. 2021) (Jacobson, Hunter et al. 2020); (Jacoby, Bielorai et al. 2018); (Kebriaei, Singh et al. 2016); (Kochenderfer, Dudley et al. 2015); (Maude, Laetsch et al. 2018); (Mian, Wei et al. 2021); (Nastoupil, Jain et al. 2020); (Turtle, Hanafi et al. 2016, Neelapu, Locke et al. 2017, Schuster, Svoboda et al. 2017, Turtle, Hay et al. 2017, Park, Rivière et al. 2018, Schuster, Bishop et al. 2019, Pasquini, Hu et al. 2020, Sesques, Ferrant et al. 2020, Wang, Munoz et al. 2020, Zhou, Tu et al. 2020, Shah, Bishop et al. 2021, Tan, Pan et al. 2021, Wang, Wang et al. 2021, Ying, Yang et al. 2021) |
| Age | | | | |
| <18 | 0.62 | 0.41-0.80 | 73 | (Curran, Margossian et al. 2019); (Gardner, Finney et al. 2017, Ghorashian, Kramer et al. 2019); (Jacoby, Bielorai et al. 2018, Maude, Laetsch et al. 2018); (Turtle, Hanafi et al. 2016, Neelapu, Locke et al. 2017, Schuster, Svoboda et al. 2017, Turtle, Hay et al. 2017, Park, Rivière et al. 2018, Schuster, Bishop et al. 2019, Pasquini, Hu et al. 2020, Sesques, Ferrant et al. 2020, Wang, Munoz et al. 2020, Zhou, Tu et al. 2020, Shah, Bishop et al. 2021, Tan, Pan et al. 2021, Wang, Wang et al. 2021, Ying, Yang et al. 2021) |
| >18 | 0.60 | 0.52-0.67 | 88 | (Abramson, Palomba et al. 2020, Baird, Epstein et al. 2021) (Benjamin, Graham et al. 2020) (Cappell, Sherry et al. 2020, Chen, Zhang et al. 2020) (Enblad, Karlsson et al. 2018) (Frey, Gill et al. 2020) (Gu, Liu et al. 2020) (Iacoboni, Villacampa et al. 2021) (Jacobson, Hunter et al. 2020) (Kebriaei, Singh et al. 2016, Jacoby, Bielorai et al. 2018)(Kebriaei, Singh et al. 2016, Jacoby, Bielorai et al. 2018)(Kebriaei, Singh et al. 2016, Jacoby, Bielorai et al. 2018)(Kebriaei, Singh et al. 2016, Jacoby, Bielorai et al. 2018)(Kebriaei, Singh et al. 2016, Jacoby, Bielorai et al. 2018)(Kebriaei, Singh et al. 2016, Jacoby, Bielorai et al. 2018)(Kebriaei, Singh et al. 2016, Jacoby, Bielorai et al. 2018)(Kebriaei, Singh et al. 2016, Jacoby, Bielorai et al. 2018)(Kebriaei, Singh et al. 2016, Jacoby, Bielorai et al. 2018)(Kebriaei, Singh et al. 2016, Jacoby, Bielorai et al. 2018)(Kebriaei, Singh et al. 2016, Jacoby, Bielorai et al. 2018)(Kebriaei, Singh et al. 2016, Jacoby, Bielorai et al. 2018)(Kebriaei, Singh et al. 2016, Jacoby, Bielorai et al. 2018)(Kebriaei, Singh et al. 2016, Jacoby, Bielorai et al. 2018)(Kebriaei, Singh et al. 2016, Jacoby, Bielorai et al. 2018)(Kebriaei, Singh et al. 2016, Jacoby, Bielorai et al. 2018) (Kochenderfer, Dudley et al. 2015) (Mian, Wei et al. 2021) (Nastoupil, Jain et al. 2020) (Turtle, Hanafi et al. 2016, Neelapu, Locke et al. 2017, Schuster, Svoboda et al. 2017, Turtle, Hay et al. 2017, Park, Rivière et al. 2018, Schuster, Bishop et al. 2019, Pasquini, Hu et al. 2020, Sesques, Ferrant et al. 2020, Wang, Munoz et al. 2020, Zhou, Tu et al. 2020, Shah, Bishop et al. 2021, Tan, Pan et al. 2021, Wang, Wang et al. 2021, Ying, Yang et al. 2021) |
| Interleukin used for  CAR T cell expansion | | | | |
| IL-2 | 0.56 | 0.45-0.66 | 86 | (Kochenderfer, Dudley et al. 2015, Turtle, Hanafi et al. 2016, Neelapu, Locke et al. 2017, Turtle, Hay et al. 2017, Enblad, Karlsson et al. 2018, Jacoby, Bielorai et al. 2018, Benjamin, Graham et al. 2020, Cappell, Sherry et al. 2020, Chen, Zhang et al. 2020, Gu, Liu et al. 2020, Jacobson, Hunter et al. 2020, Nastoupil, Jain et al. 2020, Sesques, Ferrant et al. 2020, Wang, Munoz et al. 2020, Baird, Epstein et al. 2021, Mian, Wei et al. 2021, Shah, Bishop et al. 2021, Tan, Pan et al. 2021) |
| Others/ Not mentioned | 0.63 | 0.50-0.75 | 91 | (Schuster, Svoboda et al. 2017, Maude, Laetsch et al. 2018, Park, Rivière et al. 2018, Curran, Margossian et al. 2019, Ghorashian, Kramer et al. 2019, Schuster, Bishop et al. 2019, Abramson, Palomba et al. 2020, Pasquini, Hu et al. 2020, Zhou, Tu et al. 2020, Iacoboni, Villacampa et al. 2021, Ying, Yang et al. 2021) |
| CAR T Cells Activation  Method | | | | |
| Anti-CD3 mAb | 0.60 | 0.45-0.74 | 86 | (Kochenderfer, Dudley et al. 2015, Kebriaei, Singh et al. 2016, Enblad, Karlsson et al. 2018, Jacoby, Bielorai et al. 2018, Schuster, Bishop et al. 2019, Cappell, Sherry et al. 2020, Sesques, Ferrant et al. 2020, Wang, Munoz et al. 2020, Shah, Bishop et al. 2021, Wang, Wang et al. 2021) |
| Anti-CD3/CD28 beads | 0.60 | 0.52-0.68 | 86 | (Turtle, Hanafi et al. 2016, Gardner, Finney et al. 2017, Neelapu, Locke et al. 2017, Schuster, Svoboda et al. 2017, Turtle, Hay et al. 2017, Maude, Laetsch et al. 2018, Park, Rivière et al. 2018, Curran, Margossian et al. 2019, Ghorashian, Kramer et al. 2019, Abramson, Palomba et al. 2020, Benjamin, Graham et al. 2020, Chen, Zhang et al. 2020, Frey, Gill et al. 2020, Gu, Liu et al. 2020, Jacobson, Hunter et al. 2020, Nastoupil, Jain et al. 2020, Pasquini, Hu et al. 2020, Sesques, Ferrant et al. 2020, Zhou, Tu et al. 2020, Baird, Epstein et al. 2021, Iacoboni, Villacampa et al. 2021, Mian, Wei et al. 2021, Tan, Pan et al. 2021, Ying, Yang et al. 2021) |
| Number of CAR T cells  injected/ kg | | | | |
| 1 to 4,9x10e6 | 0.60 | 0.50-0.69 | 85 | (Turtle, Hanafi et al. 2016, Gardner, Finney et al. 2017, Neelapu, Locke et al. 2017, Turtle, Hay et al. 2017, Jacoby, Bielorai et al. 2018, Maude, Laetsch et al. 2018, Park, Rivière et al. 2018, Curran, Margossian et al. 2019, Ghorashian, Kramer et al. 2019, Cappell, Sherry et al. 2020, Jacobson, Hunter et al. 2020, Nastoupil, Jain et al. 2020, Sesques, Ferrant et al. 2020, Wang, Munoz et al. 2020, Baird, Epstein et al. 2021, Mian, Wei et al. 2021, Shah, Bishop et al. 2021, Tan, Pan et al. 2021, Wang, Wang et al. 2021) |
| 5x10e6 to 9,9x10e7 | 0.58 | 0.21-0.88 | 66 | (Kochenderfer, Dudley et al. 2015, Schuster, Svoboda et al. 2017, Gu, Liu et al. 2020) |
| ≥10e8 | 0.56 | 0.25-0.83 | 94 | (Kebriaei, Singh et al. 2016, Enblad, Karlsson et al. 2018, Schuster, Bishop et al. 2019, Iacoboni, Villacampa et al. 2021, Ying, Yang et al. 2021) |
| Variable | 0.64 | 0.42-0.81 | 90 | (Abramson, Palomba et al. 2020, Benjamin, Graham et al. 2020, Frey, Gill et al. 2020, Pasquini, Hu et al. 2020) |
| Number of CAR T cells  infusions | | | | |
| One infusion | 0.61 | 0.52-0.69 | 88 | (Kochenderfer, Dudley et al. 2015, Kebriaei, Singh et al. 2016, Turtle, Hanafi et al. 2016, Neelapu, Locke et al. 2017, Schuster, Svoboda et al. 2017, Turtle, Hay et al. 2017, Enblad, Karlsson et al. 2018, Jacoby, Bielorai et al. 2018, Maude, Laetsch et al. 2018, Ghorashian, Kramer et al. 2019, Schuster, Bishop et al. 2019, Cappell, Sherry et al. 2020, Chen, Zhang et al. 2020, Gu, Liu et al. 2020, Jacobson, Hunter et al. 2020, Nastoupil, Jain et al. 2020, Pasquini, Hu et al. 2020, Sesques, Ferrant et al. 2020, Wang, Munoz et al. 2020, Zhou, Tu et al. 2020, Baird, Epstein et al. 2021, Iacoboni, Villacampa et al. 2021, Mian, Wei et al. 2021, Shah, Bishop et al. 2021, Tan, Pan et al. 2021, Wang, Wang et al. 2021, Ying, Yang et al. 2021) |
| Two infusions | 0.70 | 0.55-0.82 | NA | (Gardner, Finney et al. 2017) |
| ≥3 infusions | 0.58 | 0.29-0.83 | 66 | (Park, Rivière et al. 2018, Curran, Margossian et al. 2019, Frey, Gill et al. 2020) |
| Variable | 0.57 | 0.23-0.85 | 66 | (Abramson, Palomba et al. 2020, Benjamin, Graham et al. 2020) |
| Cell population transduced  or transfected with the CAR | | | | |
| PBMCs | 0.61 | 0.53-0.73 | 86 | (Kochenderfer, Dudley et al. 2015, Kebriaei, Singh et al. 2016, Neelapu, Locke et al. 2017, Schuster, Svoboda et al. 2017, Enblad, Karlsson et al. 2018, Jacoby, Bielorai et al. 2018, Maude, Laetsch et al. 2018, Park, Rivière et al. 2018, Curran, Margossian et al. 2019, Ghorashian, Kramer et al. 2019, Schuster, Bishop et al. 2019, Cappell, Sherry et al. 2020, Chen, Zhang et al. 2020, Frey, Gill et al. 2020, Gu, Liu et al. 2020, Jacobson, Hunter et al. 2020, Nastoupil, Jain et al. 2020, Pasquini, Hu et al. 2020, Sesques, Ferrant et al. 2020, Wang, Munoz et al. 2020, Zhou, Tu et al. 2020, Iacoboni, Villacampa et al. 2021, Mian, Wei et al. 2021, Shah, Bishop et al. 2021, Tan, Pan et al. 2021, Wang, Wang et al. 2021) |
| CD4/CD8 1:1 or CD8+ only or specific subsets | 0.55 | 0.35-0.73 | 86 | (Turtle, Hanafi et al. 2016, Gardner, Finney et al. 2017, Turtle, Hay et al. 2017, Abramson, Palomba et al. 2020, Baird, Epstein et al. 2021, Ying, Yang et al. 2021) |
| Anti-CD19 CAR Type (Name) | | | | |
| Axicabtagene citoleucel (Yescarta) (Axi-cel) KTE-C19 | 0.68 | 0.59-0.77 | 80 | (Neelapu, Locke et al. 2017, Jacoby, Bielorai et al. 2018, Park, Rivière et al. 2018, Cappell, Sherry et al. 2020, Jacobson, Hunter et al. 2020, Nastoupil, Jain et al. 2020, Sesques, Ferrant et al. 2020, Wang, Munoz et al. 2020, Baird, Epstein et al. 2021, Mian, Wei et al. 2021) |
| Tisagenlecleucel (CTL019) (Kymriah) (Tisa-cel) | 0.61 | 0.42-0.76 | 92 | (Kochenderfer, Dudley et al. 2015, Schuster, Svoboda et al. 2017, Maude, Laetsch et al. 2018, Schuster, Bishop et al. 2019, Chen, Zhang et al. 2020, Frey, Gill et al. 2020, Pasquini, Hu et al. 2020, Sesques, Ferrant et al. 2020, Iacoboni, Villacampa et al. 2021, Tan, Pan et al. 2021) |
| JCAR014 | 0.35 | 0.00-1.00 | 92 | (Turtle, Hanafi et al. 2016, Turtle, Hay et al. 2017) |
| Others | 0.57 | 0.52-0.62 | 40 | (Gardner, Finney et al. 2017, Enblad, Karlsson et al. 2018, Ghorashian, Kramer et al. 2019, Abramson, Palomba et al. 2020, Benjamin, Graham et al. 2020, Gu, Liu et al. 2020, Zhou, Tu et al. 2020, Shah, Bishop et al. 2021, Wang, Wang et al. 2021, Ying, Yang et al. 2021) |
| CAR Hinge  Domain | | | | |
| CD8 | 0.59 | 0.46-0.71 | 89 | (Kochenderfer, Dudley et al. 2015, Schuster, Svoboda et al. 2017, Maude, Laetsch et al. 2018, Ghorashian, Kramer et al. 2019, Schuster, Bishop et al. 2019, Benjamin, Graham et al. 2020, Chen, Zhang et al. 2020, Frey, Gill et al. 2020, Gu, Liu et al. 2020, Pasquini, Hu et al. 2020, Sesques, Ferrant et al. 2020, Zhou, Tu et al. 2020, Iacoboni, Villacampa et al. 2021, Tan, Pan et al. 2021, Wang, Wang et al. 2021) |
| CD28 | 0.65 | 0.55-0.74 | 81 | (Neelapu, Locke et al. 2017, Enblad, Karlsson et al. 2018, Jacoby, Bielorai et al. 2018, Park, Rivière et al. 2018, Cappell, Sherry et al. 2020, Jacobson, Hunter et al. 2020, Nastoupil, Jain et al. 2020, Sesques, Ferrant et al. 2020, Wang, Munoz et al. 2020, Baird, Epstein et al. 2021, Mian, Wei et al. 2021, Shah, Bishop et al. 2021) |
| IgG4 | 0.50 | 0.32-0.59 | 84 | (Kebriaei, Singh et al. 2016, Turtle, Hanafi et al. 2016, Turtle, Hay et al. 2017, Curran, Margossian et al. 2019, Abramson, Palomba et al. 2020, Ying, Yang et al. 2021) |
| CAR Transmembrane  Domain | | | | |
| CD8alpha | 0.59 | 0-45-0.72 | 90 | (Kochenderfer, Dudley et al. 2015, Schuster, Svoboda et al. 2017, Maude, Laetsch et al. 2018, Ghorashian, Kramer et al. 2019, Schuster, Bishop et al. 2019, Benjamin, Graham et al. 2020, Chen, Zhang et al. 2020, Frey, Gill et al. 2020, Pasquini, Hu et al. 2020, Sesques, Ferrant et al. 2020, Iacoboni, Villacampa et al. 2021, Tan, Pan et al. 2021, Wang, Wang et al. 2021) |
| CD28 | 0.61 | 0.51-0.70 | 85 | (Kebriaei, Singh et al. 2016, Turtle, Hanafi et al. 2016, Gardner, Finney et al. 2017, Neelapu, Locke et al. 2017, Turtle, Hay et al. 2017, Enblad, Karlsson et al. 2018, Jacoby, Bielorai et al. 2018, Park, Rivière et al. 2018, Curran, Margossian et al. 2019, Cappell, Sherry et al. 2020, Gu, Liu et al. 2020, Jacobson, Hunter et al. 2020, Nastoupil, Jain et al. 2020, Sesques, Ferrant et al. 2020, Wang, Munoz et al. 2020, Zhou, Tu et al. 2020, Baird, Epstein et al. 2021, Mian, Wei et al. 2021, Shah, Bishop et al. 2021) |
| CAR Costimulatory  Domain | | | | |
| CD28 | 0.66 | 0.57-0.74 | 79 | (Kebriaei, Singh et al. 2016, Neelapu, Locke et al. 2017, Jacoby, Bielorai et al. 2018, Park, Rivière et al. 2018, Curran, Margossian et al. 2019, Cappell, Sherry et al. 2020, Jacobson, Hunter et al. 2020, Nastoupil, Jain et al. 2020, Sesques, Ferrant et al. 2020, Wang, Munoz et al. 2020, Baird, Epstein et al. 2021, Mian, Wei et al. 2021, Shah, Bishop et al. 2021) |
| 4-1BB | 0.56 | 0.45-0.66 | 89 | (Kochenderfer, Dudley et al. 2015, Turtle, Hanafi et al. 2016, Gardner, Finney et al. 2017, Schuster, Svoboda et al. 2017, Turtle, Hay et al. 2017, Maude, Laetsch et al. 2018, Ghorashian, Kramer et al. 2019, Schuster, Bishop et al. 2019, Abramson, Palomba et al. 2020, Benjamin, Graham et al. 2020, Chen, Zhang et al. 2020, Frey, Gill et al. 2020, Gu, Liu et al. 2020, Pasquini, Hu et al. 2020, Sesques, Ferrant et al. 2020, Iacoboni, Villacampa et al. 2021, Tan, Pan et al. 2021, Wang, Wang et al. 2021, Ying, Yang et al. 2021) |
| Tumor Type | | | | |
| NHL | 0.59 | 0.46-0.72 | 92 | (Turtle, Hanafi et al. 2016, Neelapu, Locke et al. 2017, Schuster, Svoboda et al. 2017, Schuster, Bishop et al. 2019, Abramson, Palomba et al. 2020, Jacobson, Hunter et al. 2020, Nastoupil, Jain et al. 2020, Sesques, Ferrant et al. 2020, Zhou, Tu et al. 2020, Baird, Epstein et al. 2021, Iacoboni, Villacampa et al. 2021, Mian, Wei et al. 2021, Ying, Yang et al. 2021) |
| ALL | 0.57 | 0.45-0.68 | 67 | (Gardner, Finney et al. 2017, Jacoby, Bielorai et al. 2018, Maude, Laetsch et al. 2018, Park, Rivière et al. 2018, Curran, Margossian et al. 2019, Ghorashian, Kramer et al. 2019, Benjamin, Graham et al. 2020, Chen, Zhang et al. 2020, Gu, Liu et al. 2020, Shah, Bishop et al. 2021, Tan, Pan et al. 2021, Wang, Wang et al. 2021) |

**Supplementary References**

Abramson, J. S., M. L. Palomba, L. I. Gordon, M. A. Lunning, M. Wang, J. Arnason, A. Mehta, E. Purev, D. G. Maloney, C. Andreadis, A. Sehgal, S. R. Solomon, N. Ghosh, T. M. Albertson, J. Garcia, A. Kostic, M. Mallaney, K. Ogasawara, K. Newhall, Y. Kim, D. Li and T. Siddiqi (2020). "Lisocabtagene maraleucel for patients with relapsed or refractory large B-cell lymphomas (TRANSCEND NHL 001): a multicentre seamless design study." Lancet **396**(10254): 839-852.

Agresti, A. and B. A. Coull (1998). "Approximate Is Better than "Exact" for Interval Estimation of Binomial Proportions." The American Statistician **52**(2): 119-126.

Baird, J. H., D. J. Epstein, J. S. Tamaresis, Z. Ehlinger, J. Y. Spiegel, J. Craig, G. K. Claire, M. J. Frank, L. Muffly, P. Shiraz, E. Meyer, S. Arai, J. W. Brown, L. Johnston, R. Lowsky, R. S. Negrin, A. R. Rezvani, W. K. Weng, T. Latchford, B. Sahaf, C. L. Mackall, D. B. Miklos and S. Sidana (2021). "Immune reconstitution and infectious complications following axicabtagene ciloleucel therapy for large B-cell lymphoma." Blood Adv **5**(1): 143-155.

Balduzzi, S., G. Rucker and G. Schwarzer (2019). "How to perform a meta-analysis with R: a practical tutorial." Evid Based Ment Health **22**(4): 153-160.

Baujat, B., C. Mahe, J. P. Pignon and C. Hill (2002). "A graphical method for exploring heterogeneity in meta-analyses: application to a meta-analysis of 65 trials." Stat Med **21**(18): 2641-2652.

Benjamin, R., C. Graham, D. Yallop, A. Jozwik, O. C. Mirci-Danicar, G. Lucchini, D. Pinner, N. Jain, H. Kantarjian, N. Boissel, M. V. Maus, M. J. Frigault, A. Baruchel, M. Mohty, A. Gianella-Borradori, F. Binlich, S. Balandraud, F. Vitry, E. Thomas, A. Philippe, S. Fouliard, S. Dupouy, I. Marchiq, M. Almena-Carrasco, N. Ferry, S. Arnould, C. Konto, P. Veys and W. Qasim (2020). "Genome-edited, donor-derived allogeneic anti-CD19 chimeric antigen receptor T cells in paediatric and adult B-cell acute lymphoblastic leukaemia: results of two phase 1 studies." Lancet **396**(10266): 1885-1894.

Cappell, K. M., R. M. Sherry, J. C. Yang, S. L. Goff, D. A. Vanasse, L. McIntyre, S. A. Rosenberg and J. N. Kochenderfer (2020). "Long-Term Follow-Up of Anti-CD19 Chimeric Antigen Receptor T-Cell Therapy." J Clin Oncol **38**(32): 3805-3815.

Chen, Y. H., X. Zhang, Y. F. Cheng, H. Chen, X. D. Mo, C. H. Yan, Y. Chen, W. Han, Y. Q. Sun, Y. Wang, X. H. Zhang, L. P. Xu, K. Y. Liu, J. Yang, J. Zhang, G. L. Zhang, Y. Shi, Y. C. Su, W. Q. Li, L. Xu, D. Song, M. Zhang, P. Lu and X. J. Huang (2020). "Long-term follow-up of CD19 chimeric antigen receptor T-cell therapy for relapsed/refractory acute lymphoblastic leukemia after allogeneic hematopoietic stem cell transplantation." Cytotherapy **22**(12): 755-761.

Curran, K. J., S. P. Margossian, N. A. Kernan, L. B. Silverman, D. A. Williams, N. Shukla, R. Kobos, C. J. Forlenza, P. Steinherz, S. Prockop, F. Boulad, B. Spitzer, M. I. Cancio, J. J. Boelens, A. L. Kung, Y. Khakoo, V. Szenes, J. H. Park, C. S. Sauter, G. Heller, X. Wang, B. Senechal, R. J. O'Reilly, I. Riviere, M. Sadelain and R. J. Brentjens (2019). "Toxicity and response after CD19-specific CAR T-cell therapy in pediatric/young adult relapsed/refractory B-ALL." Blood **134**(26): 2361-2368.

Enblad, G., H. Karlsson, G. Gammelgård, J. Wenthe, T. Lövgren, R. M. Amini, K. I. Wikstrom, M. Essand, B. Savoldo, H. Hallböök, M. Höglund, G. Dotti, M. K. Brenner, H. Hagberg and A. Loskog (2018). "A Phase I/IIa Trial Using CD19-Targeted Third-Generation CAR T Cells for Lymphoma and Leukemia." Clin Cancer Res **24**(24): 6185-6194.

Frey, N. V., S. Gill, E. O. Hexner, S. Schuster, S. Nasta, A. Loren, J. Svoboda, E. Stadtmauer, D. J. Landsburg, A. Mato, B. L. Levine, S. F. Lacey, J. J. Melenhorst, E. Veloso, A. Gaymon, E. Pequignot, X. Shan, W. T. Hwang, C. H. June and D. L. Porter (2020). "Long-Term Outcomes From a Randomized Dose Optimization Study of Chimeric Antigen Receptor Modified T Cells in Relapsed Chronic Lymphocytic Leukemia." J Clin Oncol **38**(25): 2862-2871.

Gardner, R. A., O. Finney, C. Annesley, H. Brakke, C. Summers, K. Leger, M. Bleakley, C. Brown, S. Mgebroff, K. S. Kelly-Spratt, V. Hoglund, C. Lindgren, A. P. Oron, D. Li, S. R. Riddell, J. R. Park and M. C. Jensen (2017). "Intent-to-treat leukemia remission by CD19 CAR T cells of defined formulation and dose in children and young adults." Blood **129**(25): 3322-3331.

Ghorashian, S., A. M. Kramer, S. Onuoha, G. Wright, J. Bartram, R. Richardson, S. J. Albon, J. Casanovas-Company, F. Castro, B. Popova, K. Villanueva, J. Yeung, W. Vetharoy, A. Guvenel, P. A. Wawrzyniecka, L. Mekkaoui, G. W. Cheung, D. Pinner, J. Chu, G. Lucchini, J. Silva, O. Ciocarlie, A. Lazareva, S. Inglott, K. C. Gilmour, G. Ahsan, M. Ferrari, S. Manzoor, K. Champion, T. Brooks, A. Lopes, A. Hackshaw, F. Farzaneh, R. Chiesa, K. Rao, D. Bonney, S. Samarasinghe, N. Goulden, A. Vora, P. Veys, R. Hough, R. Wynn, M. A. Pule and P. J. Amrolia (2019). "Enhanced CAR T cell expansion and prolonged persistence in pediatric patients with ALL treated with a low-affinity CD19 CAR." Nat Med **25**(9): 1408-1414.

Gu, R., F. Liu, D. Zou, Y. Xu, Y. Lu, B. Liu, W. Liu, X. Chen, K. Liu, Y. Guo, X. Gong, R. Lv, C. Zhou, M. Zhong, H. Wang, H. Wei, Y. Mi, L. Qiu, L. Lv, M. Wang, Y. Wang, X. Zhu and J. Wang (2020). "Efficacy and safety of CD19 CAR T constructed with a new anti-CD19 chimeric antigen receptor in relapsed or refractory acute lymphoblastic leukemia." J Hematol Oncol **13**(1): 122.

Harrer, M. (2022). Doing meta-analysis with R : a hands-on guide. Boca Raton, CRC Press,**:** 1 online resource.

Harrer, M., J. Apolinario-Hagen, L. Fritsche, M. Druge, L. Krings, K. Beck, C. Salewski, A. C. Zarski, D. Lehr, H. Baumeister and D. D. Ebert (2019). "Internet- and App-Based Stress Intervention for Distance-Learning Students With Depressive Symptoms: Protocol of a Randomized Controlled Trial." Front Psychiatry **10**: 361.

Hartung, J. and G. Knapp (2001). "On tests of the overall treatment effect in meta-analysis with normally distributed responses." Stat Med **20**(12): 1771-1782.

Higgins, J. P. and S. G. Thompson (2002). "Quantifying heterogeneity in a meta-analysis." Stat Med **21**(11): 1539-1558.

Iacoboni, G., G. Villacampa, N. Martinez-Cibrian, R. Bailén, L. Lopez Corral, J. M. Sanchez, M. Guerreiro, A. C. Caballero, A. Mussetti, J. M. Sancho, R. Hernani, P. Abrisqueta, C. Solano, A. Sureda, J. Briones, A. Martin Garcia-Sancho, M. Kwon, J. L. Reguera-Ortega and P. Barba (2021). "Real-world evidence of tisagenlecleucel for the treatment of relapsed or refractory large B-cell lymphoma." Cancer Med **10**(10): 3214-3223.

Jacobson, C. A., B. D. Hunter, R. Redd, S. J. Rodig, P. H. Chen, K. Wright, M. Lipschitz, J. Ritz, Y. Kamihara, P. Armand, S. Nikiforow, M. Rogalski, J. Maakaron, S. Jaglowski, M. V. Maus, Y. B. Chen, J. S. Abramson, J. Kline, E. Budde, A. Herrera, M. Mei, J. B. Cohen, S. D. Smith, D. G. Maloney, A. K. Gopal, M. J. Frigault and U. H. Acharya (2020). "Axicabtagene Ciloleucel in the Non-Trial Setting: Outcomes and Correlates of Response, Resistance, and Toxicity." J Clin Oncol **38**(27): 3095-3106.

Jacoby, E., B. Bielorai, A. Avigdor, O. Itzhaki, D. Hutt, V. Nussboim, A. Meir, A. Kubi, M. Levy, D. Zikich, L. A. Zeltzer, K. Brezinger, J. Schachter, A. Nagler, M. J. Besser and A. Toren (2018). "Locally produced CD19 CAR T cells leading to clinical remissions in medullary and extramedullary relapsed acute lymphoblastic leukemia." Am J Hematol **93**(12): 1485-1492.

Kebriaei, P., H. Singh, M. H. Huls, M. J. Figliola, R. Bassett, S. Olivares, B. Jena, M. J. Dawson, P. R. Kumaresan, S. Su, S. Maiti, J. Dai, B. Moriarity, M. A. Forget, V. Senyukov, A. Orozco, T. Liu, J. McCarty, R. N. Jackson, J. S. Moyes, G. Rondon, M. Qazilbash, S. Ciurea, A. Alousi, Y. Nieto, K. Rezvani, D. Marin, U. Popat, C. Hosing, E. J. Shpall, H. Kantarjian, M. Keating, W. Wierda, K. A. Do, D. A. Largaespada, D. A. Lee, P. B. Hackett, R. E. Champlin and L. J. Cooper (2016). "Phase I trials using Sleeping Beauty to generate CD19-specific CAR T cells." J Clin Invest **126**(9): 3363-3376.

Kochenderfer, J. N., M. E. Dudley, S. H. Kassim, R. P. Somerville, R. O. Carpenter, M. Stetler-Stevenson, J. C. Yang, G. Q. Phan, M. S. Hughes, R. M. Sherry, M. Raffeld, S. Feldman, L. Lu, Y. F. Li, L. T. Ngo, A. Goy, T. Feldman, D. E. Spaner, M. L. Wang, C. C. Chen, S. M. Kranick, A. Nath, D. A. Nathan, K. E. Morton, M. A. Toomey and S. A. Rosenberg (2015). "Chemotherapy-refractory diffuse large B-cell lymphoma and indolent B-cell malignancies can be effectively treated with autologous T cells expressing an anti-CD19 chimeric antigen receptor." J Clin Oncol **33**(6): 540-549.

Maude, S. L., T. W. Laetsch, J. Buechner, S. Rives, M. Boyer, H. Bittencourt, P. Bader, M. R. Verneris, H. E. Stefanski, G. D. Myers, M. Qayed, B. De Moerloose, H. Hiramatsu, K. Schlis, K. L. Davis, P. L. Martin, E. R. Nemecek, G. A. Yanik, C. Peters, A. Baruchel, N. Boissel, F. Mechinaud, A. Balduzzi, J. Krueger, C. H. June, B. L. Levine, P. Wood, T. Taran, M. Leung, K. T. Mueller, Y. Zhang, K. Sen, D. Lebwohl, M. A. Pulsipher and S. A. Grupp (2018). "Tisagenlecleucel in Children and Young Adults with B-Cell Lymphoblastic Leukemia." N Engl J Med **378**(5): 439-448.

Mian, A., W. Wei, A. M. Winter, J. Khouri, D. Jagadeesh, F. Anwer, A. T. Gerds, R. M. Dean, R. Sobecks, B. Pohlman, B. K. Hamilton, N. S. Majhail and B. T. Hill (2021). "Outcomes and factors impacting use of axicabtagene ciloleucel in patients with relapsed or refractory large B-cell lymphoma: results from an intention-to-treat analysis." Leuk Lymphoma **62**(6): 1344-1352.

Nastoupil, L. J., M. D. Jain, L. Feng, J. Y. Spiegel, A. Ghobadi, Y. Lin, S. Dahiya, M. Lunning, L. Lekakis, P. Reagan, O. Oluwole, J. McGuirk, A. Deol, A. R. Sehgal, A. Goy, B. T. Hill, K. Vu, C. Andreadis, J. Munoz, J. Westin, J. C. Chavez, A. Cashen, N. N. Bennani, A. P. Rapoport, J. M. Vose, D. B. Miklos, S. S. Neelapu and F. L. Locke (2020). "Standard-of-Care Axicabtagene Ciloleucel for Relapsed or Refractory Large B-Cell Lymphoma: Results From the US Lymphoma CAR T Consortium." J Clin Oncol **38**(27): 3119-3128.

Neelapu, S. S., F. L. Locke, N. L. Bartlett, L. J. Lekakis, D. B. Miklos, C. A. Jacobson, I. Braunschweig, O. O. Oluwole, T. Siddiqi, Y. Lin, J. M. Timmerman, P. J. Stiff, J. W. Friedberg, I. W. Flinn, A. Goy, B. T. Hill, M. R. Smith, A. Deol, U. Farooq, P. McSweeney, J. Munoz, I. Avivi, J. E. Castro, J. R. Westin, J. C. Chavez, A. Ghobadi, K. V. Komanduri, R. Levy, E. D. Jacobsen, T. E. Witzig, P. Reagan, A. Bot, J. Rossi, L. Navale, Y. Jiang, J. Aycock, M. Elias, D. Chang, J. Wiezorek and W. Y. Go (2017). "Axicabtagene Ciloleucel CAR T-Cell Therapy in Refractory Large B-Cell Lymphoma." N Engl J Med **377**(26): 2531-2544.

Page, M. J., J. E. McKenzie, P. M. Bossuyt, I. Boutron, T. C. Hoffmann, C. D. Mulrow, L. Shamseer, J. M. Tetzlaff, E. A. Akl, S. E. Brennan, R. Chou, J. Glanville, J. M. Grimshaw, A. Hrobjartsson, M. M. Lalu, T. Li, E. W. Loder, E. Mayo-Wilson, S. McDonald, L. A. McGuinness, L. A. Stewart, J. Thomas, A. C. Tricco, V. A. Welch, P. Whiting and D. Moher (2021). "The PRISMA 2020 statement: An updated guideline for reporting systematic reviews." J Clin Epidemiol **134**: 178-189.

Park, J. H., I. Rivière, M. Gonen, X. Wang, B. Sénéchal, K. J. Curran, C. Sauter, Y. Wang, B. Santomasso, E. Mead, M. Roshal, P. Maslak, M. Davila, R. J. Brentjens and M. Sadelain (2018). "Long-Term Follow-up of CD19 CAR Therapy in Acute Lymphoblastic Leukemia." N Engl J Med **378**(5): 449-459.

Pasquini, M. C., Z. H. Hu, K. Curran, T. Laetsch, F. Locke, R. Rouce, M. A. Pulsipher, C. L. Phillips, A. Keating, M. J. Frigault, D. Salzberg, S. Jaglowski, J. P. Sasine, J. Rosenthal, M. Ghosh, D. Landsburg, S. Margossian, P. L. Martin, M. K. Kamdar, P. Hematti, S. Nikiforow, C. Turtle, M. A. Perales, P. Steinert, M. M. Horowitz, A. Moskop, L. Pacaud, L. Yi, R. Chawla, E. Bleickardt and S. Grupp (2020). "Real-world evidence of tisagenlecleucel for pediatric acute lymphoblastic leukemia and non-Hodgkin lymphoma." Blood Adv **4**(21): 5414-5424.

Schuster, S. J., M. R. Bishop, C. S. Tam, E. K. Waller, P. Borchmann, J. P. McGuirk, U. Jäger, S. Jaglowski, C. Andreadis, J. R. Westin, I. Fleury, V. Bachanova, S. R. Foley, P. J. Ho, S. Mielke, J. M. Magenau, H. Holte, S. Pantano, L. B. Pacaud, R. Awasthi, J. Chu, Ö. Anak, G. Salles and R. T. Maziarz (2019). "Tisagenlecleucel in Adult Relapsed or Refractory Diffuse Large B-Cell Lymphoma." N Engl J Med **380**(1): 45-56.

Schuster, S. J., J. Svoboda, E. A. Chong, S. D. Nasta, A. R. Mato, Ö. Anak, J. L. Brogdon, I. Pruteanu-Malinici, V. Bhoj, D. Landsburg, M. Wasik, B. L. Levine, S. F. Lacey, J. J. Melenhorst, D. L. Porter and C. H. June (2017). "Chimeric Antigen Receptor T Cells in Refractory B-Cell Lymphomas." N Engl J Med **377**(26): 2545-2554.

Sesques, P., E. Ferrant, V. Safar, F. Wallet, J. Tordo, A. Dhomps, L. Karlin, G. Brisou, M. Vercasson, C. Hospital-Gustem, V. Schwiertz, F. Ranchon, C. Rioufol, M. Choquet, P. Sujobert, D. Ghergus, F. Bouafia, C. Golfier, H. Lequeu, A. Lazareth, S. Novelli, P. Devic, A. Traverse Glehen, S. Viel, F. Venet, V. Mialou, O. Hequet, A. Chauchet, Y. Arkam, E. Nicolas-Virelizier, F. Peyrade, D. Cavalieri, F. Ader, H. Ghesquières, G. Salles and E. Bachy (2020). "Commercial anti-CD19 CAR T cell therapy for patients with relapsed/refractory aggressive B cell lymphoma in a European center." Am J Hematol **95**(11): 1324-1333.

Shadish, W. R., L. V. Hedges and J. E. Pustejovsky (2014). "Analysis and meta-analysis of single-case designs with a standardized mean difference statistic: a primer and applications." J Sch Psychol **52**(2): 123-147.

Shah, B. D., M. R. Bishop, O. O. Oluwole, A. C. Logan, M. R. Baer, W. B. Donnellan, K. M. O'Dwyer, H. Holmes, M. L. Arellano, A. Ghobadi, J. M. Pagel, Y. Lin, R. D. Cassaday, J. H. Park, M. Abedi, J. E. Castro, D. J. DeAngelo, A. K. Malone, R. Mawad, G. J. Schiller, J. M. Rossi, A. Bot, T. Shen, L. Goyal, R. K. Jain, R. Vezan and W. G. Wierda (2021). "KTE-X19 anti-CD19 CAR T-cell therapy in adult relapsed/refractory acute lymphoblastic leukemia: ZUMA-3 phase 1 results." Blood **138**(1): 11-22.

Sterne, J. A. and M. Egger (2001). "Funnel plots for detecting bias in meta-analysis: guidelines on choice of axis." J Clin Epidemiol **54**(10): 1046-1055.

Tan, Y., J. Pan, B. Deng, Z. Ling, W. Song, J. Xu, J. Duan, Z. Wang, X. Yu, A. H. Chang and X. Feng (2021). "Toxicity and effectiveness of CD19 CAR T therapy in children with high-burden central nervous system refractory B-ALL." Cancer Immunol Immunother **70**(7): 1979-1993.

Turtle, C. J., L. A. Hanafi, C. Berger, M. Hudecek, B. Pender, E. Robinson, R. Hawkins, C. Chaney, S. Cherian, X. Chen, L. Soma, B. Wood, D. Li, S. Heimfeld, S. R. Riddell and D. G. Maloney (2016). "Immunotherapy of non-Hodgkin's lymphoma with a defined ratio of CD8+ and CD4+ CD19-specific chimeric antigen receptor-modified T cells." Sci Transl Med **8**(355): 355ra116.

Turtle, C. J., K. A. Hay, L. A. Hanafi, D. Li, S. Cherian, X. Chen, B. Wood, A. Lozanski, J. C. Byrd, S. Heimfeld, S. R. Riddell and D. G. Maloney (2017). "Durable Molecular Remissions in Chronic Lymphocytic Leukemia Treated With CD19-Specific Chimeric Antigen Receptor-Modified T Cells After Failure of Ibrutinib." J Clin Oncol **35**(26): 3010-3020.

Veroniki, A. A., D. Jackson, W. Viechtbauer, R. Bender, J. Bowden, G. Knapp, O. Kuss, J. P. Higgins, D. Langan and G. Salanti (2016). "Methods to estimate the between-study variance and its uncertainty in meta-analysis." Res Synth Methods **7**(1): 55-79.

Wang, M., J. Munoz, A. Goy, F. L. Locke, C. A. Jacobson, B. T. Hill, J. M. Timmerman, H. Holmes, S. Jaglowski, I. W. Flinn, P. A. McSweeney, D. B. Miklos, J. M. Pagel, M. J. Kersten, N. Milpied, H. Fung, M. S. Topp, R. Houot, A. Beitinjaneh, W. Peng, L. Zheng, J. M. Rossi, R. K. Jain, A. V. Rao and P. M. Reagan (2020). "KTE-X19 CAR T-Cell Therapy in Relapsed or Refractory Mantle-Cell Lymphoma." N Engl J Med **382**(14): 1331-1342.

Wang, S., X. Wang, C. Ye, H. Cheng, M. Shi, W. Chen, K. Qi, G. Wang, Q. Wu, L. Zeng, Z. Li, G. Jing, J. Zheng, K. Xu and J. Cao (2021). "Humanized CD19-targeted chimeric antigen receptor T (CAR-T) cells for relapsed/refractory pediatric acute lymphoblastic leukemia." Am J Hematol **96**(5): E162-e165.

Ying, Z., H. Yang, Y. Guo, W. Li, D. Zou, D. Zhou, Z. Wang, M. Zhang, J. Wu, H. Liu, P. Zhang, S. Yang, Z. Zhou, H. Zheng, Y. Song and J. Zhu (2021). "Relmacabtagene autoleucel (relma-cel) CD19 CAR-T therapy for adults with heavily pretreated relapsed/refractory large B-cell lymphoma in China." Cancer Med **10**(3): 999-1011.

Zhou, X., S. Tu, C. Wang, R. Huang, L. Deng, C. Song, C. Yue, Y. He, J. Yang, Z. Liang, A. Wu, M. Li, W. Zhou, J. Du, Z. Guo, Y. Li, C. Jiao, Y. Liu and L. J. Chang (2020). "Phase I Trial of Fourth-Generation Anti-CD19 Chimeric Antigen Receptor T Cells Against Relapsed or Refractory B Cell Non-Hodgkin Lymphomas." Front Immunol **11**: 564099.
